# Longitudinal Multi-omics and Microbiome Meta-analysis Identify an Asymptomatic Gingival State That Links Gingivitis, Periodontitis, and Aging

**DOI:** 10.1128/mBio.03281-20

**Published:** 2021-03-09

**Authors:** Shi Huang, Tao He, Feng Yue, Xiujun Xu, Lijiang Wang, Pengfei Zhu, Fei Teng, Zheng Sun, Xiaohui Liu, Gongchao Jing, Xiaoquan Su, Lijian Jin, Jiquan Liu, Jian Xu

**Affiliations:** a Single-Cell Center, Qingdao Institute of BioEnergy and Bioprocess Technology, Chinese Academy of Sciences, Qingdao, Shandong, China; b Procter & Gamble Company, Global Oral Care Clinical Operations, Mason, Ohio, USA; c Procter & Gamble Innovation Center, Beijing, China; d School of Life Sciences, Tsinghua University, Beijing, China; e Faculty of Dentistry, The University of Hong Kong, Hong Kong SAR, China; f University of Chinese Academy of Sciences, Beijing, China; g Department of Pediatrics and Center for Microbiome Innovation at Jacobs School of Engineering, University of California, San Diego, USA; Georgia Institute of Technology School of Biological Sciences

**Keywords:** gingivitis, periodontitis, microbiome, aging, disease prevention

## Abstract

Most adults experience episodes of gingivitis, which can progress to the irreversible, chronic state of periodontitis, yet roles of plaque in gingivitis onset and progression to periodontitis remain elusive. Here, we longitudinally profiled the plaque metagenome, the plaque metabolome, and salivary cytokines in 40 adults who transited from naturally occurring gingivitis (NG) to healthy gingivae (baseline) and then to experimental gingivitis (EG). During EG, rapid and consistent alterations in plaque microbiota, metabolites, and salivary cytokines emerged as early as 24 to 72 h after oral-hygiene pause, defining an asymptomatic suboptimal health (SoH) stage of the gingivae. SoH features a swift, full activation of 11 salivary cytokines but a steep synergetic decrease of plaque-derived betaine and *Rothia* spp., suggesting an anti-gum inflammation mechanism by health-promoting symbionts. Global, cross-cohort meta-analysis revealed, at SoH, a greatly elevated microbiome-based periodontitis index driven by its convergence of both taxonomical and functional profiles toward the periodontitis microbiome. Finally, post-SoH gingivitis development accelerates oral microbiota aging by over 1 year within 28 days, with *Rothia* spp. depletion and Porphyromonas gingivalis elevation as hallmarks. Thus, the microbiome-defined, transient gum SoH stage is a crucial link among gingivitis, periodontitis, and aging.

## Abstract (precis)

A significant portion of world population still fails to brush teeth daily. As a result, the majority of the global adult population is afflicted with chronic gingivitis, and if it is left untreated, some of them will eventually suffer from periodontitis.

## INTRODUCTION

Gingivitis, an inflammatory lesion of the tooth-supporting soft tissues, is one of the most common oral diseases in humans and has been a global health burden for centuries (1–5). It results from a dysregulated immunoinflammatory response which is induced by dysbiotic plaque biofilm (6). Manifesting with various clinical signs and symptoms, the condition of gingivitis is affected by both local and systemic factors (4). Notably, this inflammatory lesion can be resolved (i.e., reversed) following appropriate professional care, whereas uncontrolled gingivitis can progress to the irreversible condition periodontitis, which is characterized by destruction of tooth-supporting tissues and alveolar bone in susceptible individuals, eventually leading to tooth loss (7). As importantly, periodontal health has been widely associated with an increased risk of systemic diseases like Alzheimer’s disease, diabetes, and cardiovascular disease (8–10). Thus, promoting periodontal health and general well-being requires a thorough, mechanistic understanding of gingivitis initiation and development (11).

However, few studies have systematically characterized gingivitis development from an integrated view of both the host and oral microbiome (11). In natural human populations, gingivitis symptoms can be reversible and volatile, as numerous internally or externally imposed disturbances, including oral-hygiene practices (personal or professional), or impairment of the immune system, injury, diet, and oral state can all affect disease development and confound disease prevention and monitoring (12). Population-wide microbiome associations have unveiled the compositional shifts of plaque during gingivitis progression (13–17), and the functional potential of oral microbiome in gingivitis onset has been profiled via metagenomics and metatranscriptomic approaches (15, 18, 19). However, due to the lack of a longitudinal perspective that includes the microbiota, their metabolites, and the host immune response, the molecular mechanisms underlying gingivitis onset and progression remain ill defined (13, 19).

As for periodontitis, the irreversible and detrimental stage of gum inflammation that results from chronic, uncontrolled gingivitis, a distinct phylogenetic structure of oral microbiota in diseased hosts versus healthy ones, was revealed via 16S rRNA gene or metagenome sequencing (20–23). In particular, multiple separate cohort studies have probed the functional potential of the periodontitis-associated microbiota via metagenome (15, 18, 19, 24, 25) or metatranscriptome (26, 27) analysis. However, the inherent mechanistic link of gingivitis and periodontitis, which is crucial to clinical prevention and treatment of both diseases, has remained elusive, due to (i) the high degree of heterogeneity among hosts and the variation in experimental procedures among the microbiome-profiling endeavors and (ii) the inability to track both microbiome and host factor and interrogate their interaction over the full course of gingivitis-to-periodontitis progression within an individual.

**FIG 1:**
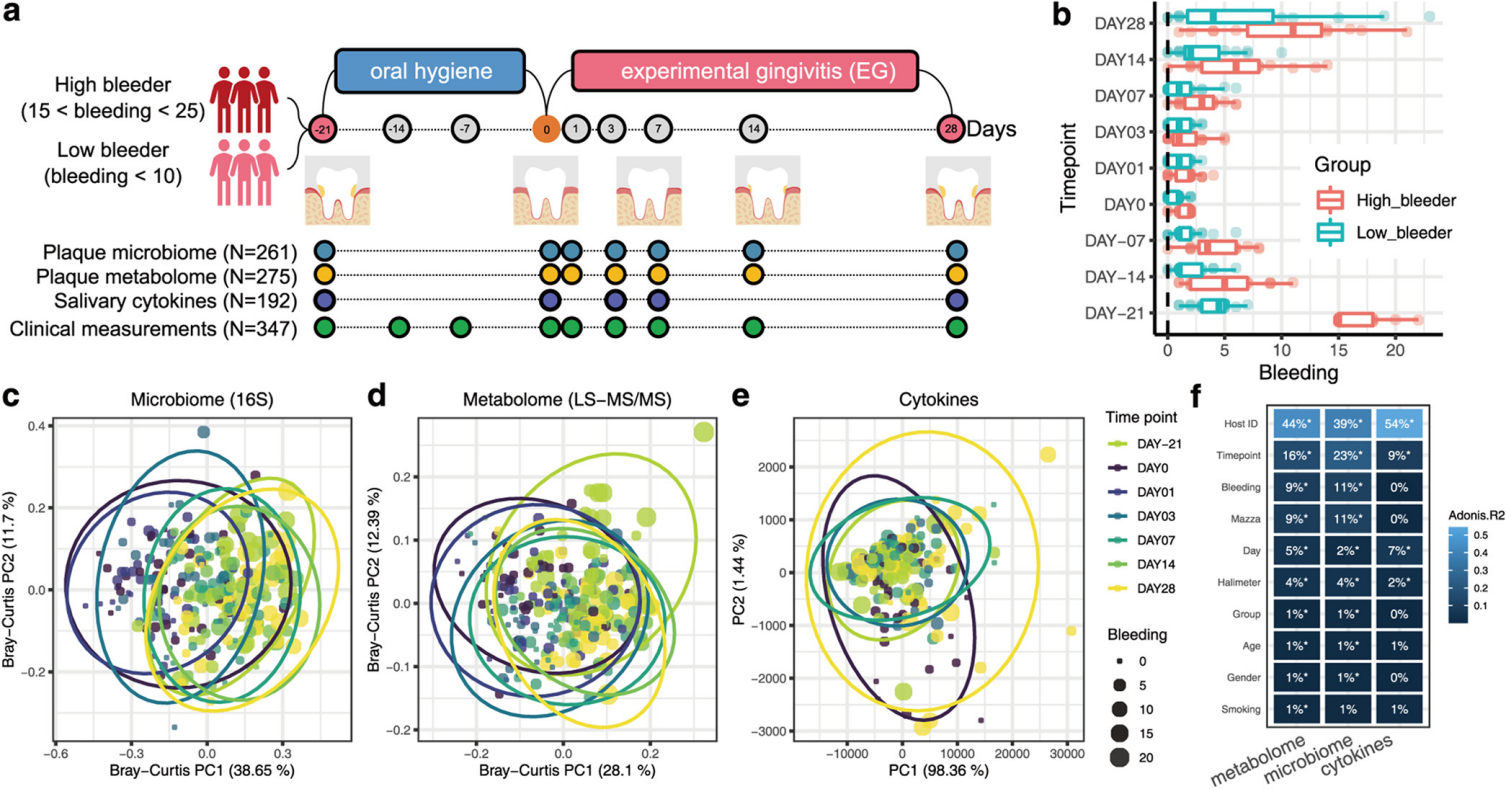
The longitudinal multi-omics landscape of gingivitis onset and progression in a human population. (a) Experimental design. Among the 40 healthy adult volunteers that participated, 20 were healthy subjects (with <10 bleeding sites), and the rest of them were unhealthy ones (bleeding sites from 15 to 25) at the start (day −21, or NG). This study yielded clinical measures (at nine time points), oral-microbiome and -metabolome data from supragingival plaque samples (at seven time points), and host immune response data from salivary samples (at five time points) for each of the 40 subjects. (b) Temporal changes in the clinical symptoms for volunteers. Boxes represent the interquartile range (IQR), and the lines inside represent the median. Whiskers denote the lowest and highest values within 1.5× IQR. (c and d) Principal-coordinate analysis (PCoA) based on the genus-level Bray-Curtis dissimilarity of (c) plaque microbiomes (16S amplicon sequencing) and (d) metabolome profiles (LC-MS/MS). (e) Principal-component analysis (PCA) of the salivary cytokine profiles. Each dot in PCoA or PCA represents a plaque or saliva sample and is included in an ellipse whose color indicates the time point. Each dot is also sized based on the severity of symptom (gum bleeding). (f) Comparing the quantitative variation in all measurements explained by the major factors. PERMANOVA shows that interindividual variation is the largest factor for all measurement types, while time and disease phenotype also capture sizable variations. FDR-corrected statistical significance: *, *P* ≤ 0.05; **, *P* ≤ 0.01; ***, *P* ≤ 0.001.

To address these key challenges, here we leveraged a longitudinal, multi-omics experimental design of human cohorts that includes personalized microbial, metabolite, and host immunoresponse profiles, to provide a high-temporal-resolution, system-level, mechanism-based landscape of the transition from periodontal health to onset of gum inflammation and eventually to gingivitis (Fig. 1). These efforts unveil, at just 24 to 72 h after pause of oral-hygiene-practice, a microbiome-defined, asymptomatic suboptimal health (SoH) stage of gingivitis, which features (i) a swift, full activation of 11 salivary cytokines, (ii) a steep, synergetic decrease of plaque-derived betaine and *Rothia* spp., and (iii) a greatly elevated microbiome-based periodontitis index driven by convergence of taxonomic and functional profiles toward the periodontitis microbiome. Intriguingly, gingivitis development greatly accelerates “oral microbiota aging” (OMA). These efforts revealed that microbiome-defined gum SoH is a crucial link between gingivitis, periodontitis, and healthy aging.

## RESULTS

### An experimentally tractable model of gingivitis onset and progression.

To control for the many confounding factors (e.g., individuality in initial gum health state or in oral-hygiene behavior) for host-microbiome dysbiosis during gingivitis (i.e., the earlier stage of periodontal disease), we designed a 40-adult cohort as an experimentally tractable model of gingivitis onset and progression (13, 16) (Fig. 1; also, see Table S1 in the supplemental material). Specifically, on day −21 (natural gingivitis [NG]), all 40 adults were assigned to one of two groups: high bleeders (15 to 25 sites of bleeding; 20 subjects) and low bleeders (0 to 10 sites of bleeding; 20 subjects) (see Materials and Methods; Fig. 1a). These hosts then underwent a rigorous oral-hygiene regimen (dental scaling) for 3 weeks, resulting in greatly reduced bleeding (median gingival bleeding of 1) on day 0 (baseline, i.e., a healthy gingival state). Next, the subjects underwent a 4-week program inducing experimental gingivitis (EG), which greatly and consistently elevated gingival bleeding, until day 28 (*P < *0.01 for gingival bleeding; i.e., the diseased state) (Fig. 1b). Notably, the between-group symptomatic difference for NG (*P = *1e−22, *t* test), the basis for the high-/low-bleeding stratification of hosts with NG (i.e., day −21), is much greater than at any of the subsequent time points (both before and after baseline) (Fig. 1b). In fact, mild or marginal differences in bleeding between high and low bleeders were observed at the seven subsequent time points (*P < *0.05, *t* test), but no such symptomatic difference was found at day 1 or 3 (*P > *0.05, *t* test). This suggested that disease severity in the natural population (i.e., NG) is not necessarily deterministic among individual hosts and that high bleeders can recover almost as rapidly and thoroughly as low bleeders if they follow a proper oral-hygiene practice.

10.1128/mBio.03281-20.6 TABLE S1 Clinical study design. This is a single-leg, examiner-blind, randomized clinical study. Forty healthy adult volunteers who met the inclusion criteria were recruited for this study. The procedures carried out at each time point during the 48-day NG-baseline-EG process are marked with an X. See Materials and Methods and Fig. 1a for additional details. Download Table S1, PDF file, 0.09 MB. Copyright © 2021 Huang et al. 2021 Huang et al. https://creativecommons.org/licenses/by/4.0/ This content is distributed under the terms of the Creative Commons Attribution 4.0 International license.

Integrated longitudinal profiles of both microbial and host immune programs were obtained by using 275 supragingival plaque samples (simultaneously for taxonomy and metabolome, via 16S rRNA amplicon sequencing and liquid chromatography-tandem mass spectrometry [LC-MS/MS]-based mass spectrometry, respectively) and 192 matching saliva samples (cytokine profile via multiplexed bead immunoassay) collected by professional dentists. The plaque microbiome, plaque metabolome, and salivary cytokines were profiled at time points that fully spanned the entire 49-day NG-baseline-EG course (from day −21 to day 28), while the transition from baseline (0 day) to the onset of EG (e.g., days 1, 3, and 7) (Fig. 1a; Fig. S1) was densely sampled, so that the tertiary interplay could be temporally monitored, especially at disease onset.

10.1128/mBio.03281-20.1 FIG S1 Key features of the 40-individual cohort. Measurements (plaque metabolome, microbiome, and salivary cytokines) available over time for each participant. Download FIG S1, PDF file, 2.9 MB. Copyright © 2021 Huang et al. 2021 Huang et al. https://creativecommons.org/licenses/by/4.0/ This content is distributed under the terms of the Creative Commons Attribution 4.0 International license.

Symptomatic severity (i.e., bleeding) contributed greatly to the first principal coordinate (PC1) in principal-coordinate analysis (PCoA) of the plaque microbiome or metabolome (Fig. 1c and d) (as revealed by the correlation between PC1 and the various factors, including number of sites of bleeding). For plaque-related measurements, although interindividual variation accounts for the majority of symptomatic variance (40 to 45%) (Fig. 1f), disease status (9 to 11%) or time point (16 to 23%) also explains much of it (Fig. 1c to f). In contrast, no significant correlation was found between bleeding and salivary cytokine profile (Fig. 1e and f). Notably, time point still explains 9% of the variation in cytokine profile (although the interindividual factor accounts for 54%); in fact, many salivary cytokines respond to gingivitis development only at the initial time points after baseline, such as day 3 or 7, but did not further increase afterwards when hosts accumulated even more bleedings. This suggests that the oral host-microbe interplay is the most intensive at the onset stages of gingivitis.

**FIG 2:**
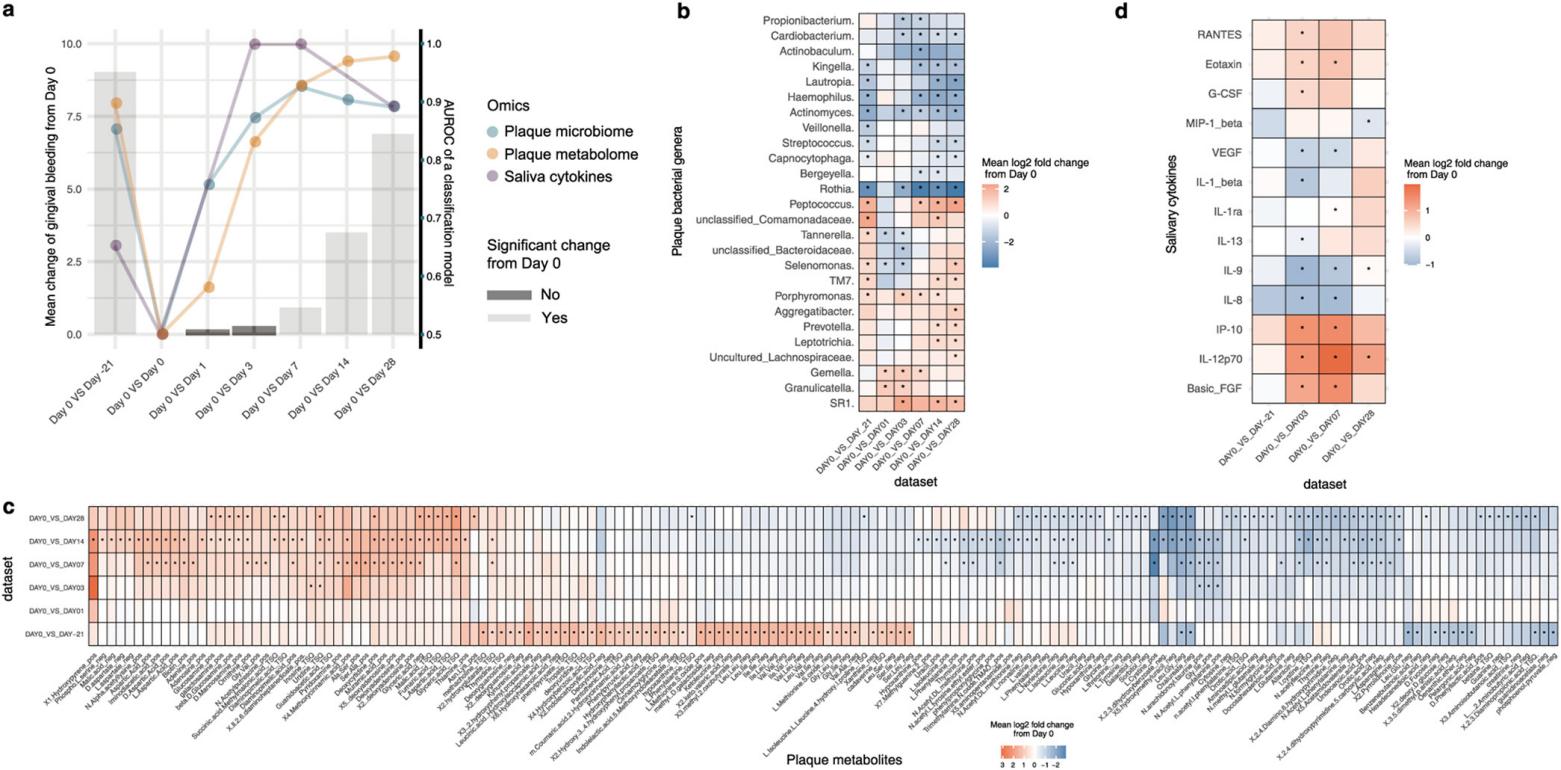
A plaque-microbiome-defined SoH stage that takes place earlier than the emergence of clinical symptoms. (a) The symptomatic change (i.e., mean bleeding difference) within hosts (*n* = 40), between each of the time points (days −21, 1, 3, 7, 14, and 28) and baseline (day 0). The colors of the bars show FDR-corrected statistical significance; specifically, days 1 to 3 are the SoH stage, when no change in clinical symptoms compared to baseline was observed within the hosts. The scatterplots show the AUROC (the *y* axis on the right) of classification models using plaque microbiota, plaque metabolome, or salivary cytokines between day 0 and each of the other time points (days −21, 1, 3, 7, 14, and 28). For panels b, c, and d, we identified molecular features from each measurement type that were differentially abundant at a given time point compared to day 0. (b) Heat map for the mean log_2_ fold change of microbial responders (threshold Bonferroni *P < *0.05) in plaque during the onset and progression of NG. (c) Heat map for the mean log_2_ fold change of both early and persistent metabolite responders (threshold Bonferroni *P < *0.05) in plaque. On the *x* axis, “pos” and “neg” indicate acquisition via positive and negative ionization modes in the nontargeted metabolomic approach, while “TSQ” indicates acquisition from the targeted metabolomic approach. (d) Heat map for the mean log_2_ fold change of cytokines at each time point (days −21, 3, 7, and 28) versus baseline (day 0). Blue denotes reduction, while red shows enrichment (versus baseline). Bonferroni-corrected statistical significance: *, *P* ≤ 0.05. The absence of an asterisk indicates no significant change.

Therefore, we hypothesized that days 1 to 3 after dental scaling represent the SoH stage (Fig. 1b). At this stage, we did not detect within-host temporal difference in clinical symptoms (i.e., from day 1 to 3 after dental scaling; *P > *0.05, paired *t* test) (Fig. 2a); however, the microbiome in the supragingival plaque and even host immune molecules might have dramatically changed due to the detrimental environmental disruptions in EG induction (i.e., poor oral hygiene).

### Swift and profound response of plaque microbiota, plaque metabolome, and salivary cytokines at SoH.

To quantitatively measure the shifts in the plaque microbiome and host immunity in the emergence of clinical symptoms, we established a unified metric to measure the temporal changes in multi-omics data from baseline to EG. Between-time-point classifiers of host gingival status were built from plaque microbiota, metabolome, and salivary cytokine profiles, via the random forests (RF) algorithm. In addition to those RF models, we employed a model accuracy metric (area under the receiver operator characteristic [AUROC]) as a proxy to quantify the temporal changes of each measurement type at each of the time points (i.e., days −21, 1, 3, 7, 14, and 28) from day 0. Furthermore, to dissect the multi-omics associations, we compared temporal changes in AUROC values of RF classifiers related to plaque microbiome, plaque metabolome, and salivary cytokines together with those from the clinical symptoms (Fig. 2a). Unexpectedly, the AUROC of RF classifiers for plaque microbiota rapidly shifted in the first 3 days (0.75 at day 1 and 0.87 at day 3) from baseline: it already resembled that of day 28 microbiota (severe gingivitis stage; AUROC = 0.89) as early as day 3 (Fig. 2a) and actually saturated after day 3. Therefore, a microbial SoH stage occurred earlier than the emergence of clinical symptoms. In concordance with plaque microbiota, the AUROC on the plaque metabolome increased quickly from 0.58 (day 1) to 0.92 (day 7) within 7 days yet did not plateau until after 14 days (AUROC = 0.97), suggesting that the plaque metabolome was persistently shifting toward a gingivitis-like state. However, the most abrupt changes in the plaque metabolome also took place in the first 3 days after dental scaling (Fig. 2a), indicating that plaque metabolome change also precedes the development of bleeding symptoms, well before they are detectable by dental health professionals. Notably, despite the concordant changes over time between the plaque microbiota and metabolome, the saturation of the AUROC of metabolome-based RF classifiers was 7 days later than that of microbiota-based classifiers (Fig. 2a), suggesting microbiome shift-dependent changes in the plaque metabolisms during gingivitis onset.

Interestingly, in the SoH stage, the immune response was even more pronounced than both the plaque microbiota and metabolome shifts (Fig. 2a). The AUROC reached almost 0.99 at day 3 to 7, while the median gingival bleeding within this period (1 for day 3 and 2 for day 7) was relatively low. In contrast, the AUROC at day −21 (i.e., naturally occurring gingivitis) and day 28 were all even lower than that in the SoH stage, while the median gingival bleeding was relatively high (8 for day 28 and 11 for day −21). This suggests that the alterations in the cytokine profiles are not necessarily associated with disease severity but are a response to the intensity or magnitude of organismal and metabolite changes in the plaque microbiome.

The longitudinal concurrent metabolomics and 16S amplicon microbial community profiling from dental plaque samples elucidated the reassembling process of supragingival plaque biofilms after dental scaling (Fig. 2a). A key question then is to identify potential microbial and metabolic factors that drive the microbial dysbiosis in the plaque. Thus, to compare the microbiome responses across different stages of disease progression, we performed differential abundance analysis on the central-log-ratio (CLR)-transformed relative abundances of each genus-level taxon between a given time point (days −21, 1, 3, 7, 14, and 28) and baseline (day 0) and compared the results across the stages of EG (Wilcoxon rank-sum test with the Bonferroni correction) (Fig. 2b). The microbial markers that were persistently enriched/depleted with gingivitis progression (such as *Porphyromonas* and *Rothia*) were termed “persistent responders,” while genera that were transiently enriched/depleted at the early stage of gingivitis progression (i.e., day 1 to 3) (such as *Gemella*) were termed “early responders.” Similarly, for the plaque metabolome, we identified a series of persistent and early responders in gingivitis development: over 50 metabolites were persistently over- or underrepresented during disease development and therefore provided a clue to pathophysiology of gingivitis (Fig. 2a and d).

Accordingly, time-resolved, differentially abundant cytokines in saliva at days −21, 3, 7, and 28 were also identified (compared to day 0) (Fig. 2c). Eleven of the 27 salivary cytokines, such as eotaxin, interleukin 5 (IL-5), MIP-1β, gamma interferon (IFN-γ), basic fibroblast growth factor (FGF), and Granulocyte colony-stimulating factor (G-CSF) altered early, within 72 h from baseline (i.e., at the SoH stage), yet did not exhibit any significant difference from baseline at later time points when gingivitis had developed (e.g., day 28, when the most severe gingivitis states were seen). In fact, the SoH stage featured prominent activation of both pro- and anti-inflammatory cytokines that stabilized in later stages of EG (Fig. 2a and c). Notably, cytokine alterations are more correlated with particular phases, such as SoH, than with gingivitis severity, which underscores the importance of high-resolution temporal views of the host-microbiome interplay.

### Integrated microbiome-metabolome dynamic profiles of oral biofilms underlying SoH.

To identify plaque microbial activities that underlie gingivitis onset and progression, we constructed a cross-measurement-type association network that incorporated both microbial taxa and metabolomes from the 261 plaque samples. To reveal trends in the data, Procrustes analysis was used to directly compare the different omics data sets (of identical internal structure) on a single principal-coordinate analysis. Overall, strong correlation between microbial taxa and metabolome of all plaque samples was observed (*r *= 0.53, *P = *0.001; Monte Carlo label permutation test) (Fig. S3a). Analysis of paired omics measurements at each of seven time points revealed remarkably high agreement between microbial taxa and metabolomics for all time points along the NG-baseline-EG course (0.6 < *r *< 0.7, *P < *0.01; Monte Carlo label permutation test) (Fig. S3b), suggesting key roles for microbe-derived metabolites in this process.

10.1128/mBio.03281-20.2 FIG S2 Temporal profiles of microbiome structure and metabolome of dental plaque unveiled the presence of a SoH stage. (a) The shifts in plaque microbiota indicate a SoH stage that takes place earlier than the shifts in clinical symptoms. (b) The fraction of microbial taxonomical markers transiently and persistently associated with gingivitis onset and progression in this 40-adult cohort. (c) The dynamics of plaque metabolome also indicate a SoH stage that emerges earlier than the manifestation of clinical symptoms. (d) The fraction of metabolite markers transiently and persistently associated with gingivitis onset and progression in this 40-adult cohort. Download FIG S2, PDF file, 0.8 MB. Copyright © 2021 Huang et al. 2021 Huang et al. https://creativecommons.org/licenses/by/4.0/ This content is distributed under the terms of the Creative Commons Attribution 4.0 International license.

10.1128/mBio.03281-20.3 FIG S3 High level of agreement between the dynamics of metabolome and microbiota along the 48-day NG-baseline-EG process as revealed by Procrustes analysis. (a) In total, 261 plaque samples were simultaneously measured for metabolomics and microbiome-structure profiles. Each point represents a plaque microbiota and was colored according to the clinical status. The arrow end of each line is connected to the 16S rRNA data of a sample, whereas the other end is connected to the corresponding plaque metabolome data. The fit of each Procrustes transformation over the first four dimensions was reported as the *P* value by 10,000 Monte Carlo label permutations. Overall, a strong correlation between microbiome and metabolome was found (*r* = 0.53). (b) A scatterplot indicates the correlation between microbiome and metabolome (with the corresponding *P* values specified) at each of seven time points along the 48-day process of gingivitis retrogression, onset, and progression. Download FIG S3, PDF file, 1.1 MB. Copyright © 2021 Huang et al. 2021 Huang et al. https://creativecommons.org/licenses/by/4.0/ This content is distributed under the terms of the Creative Commons Attribution 4.0 International license.

**FIG 3:**
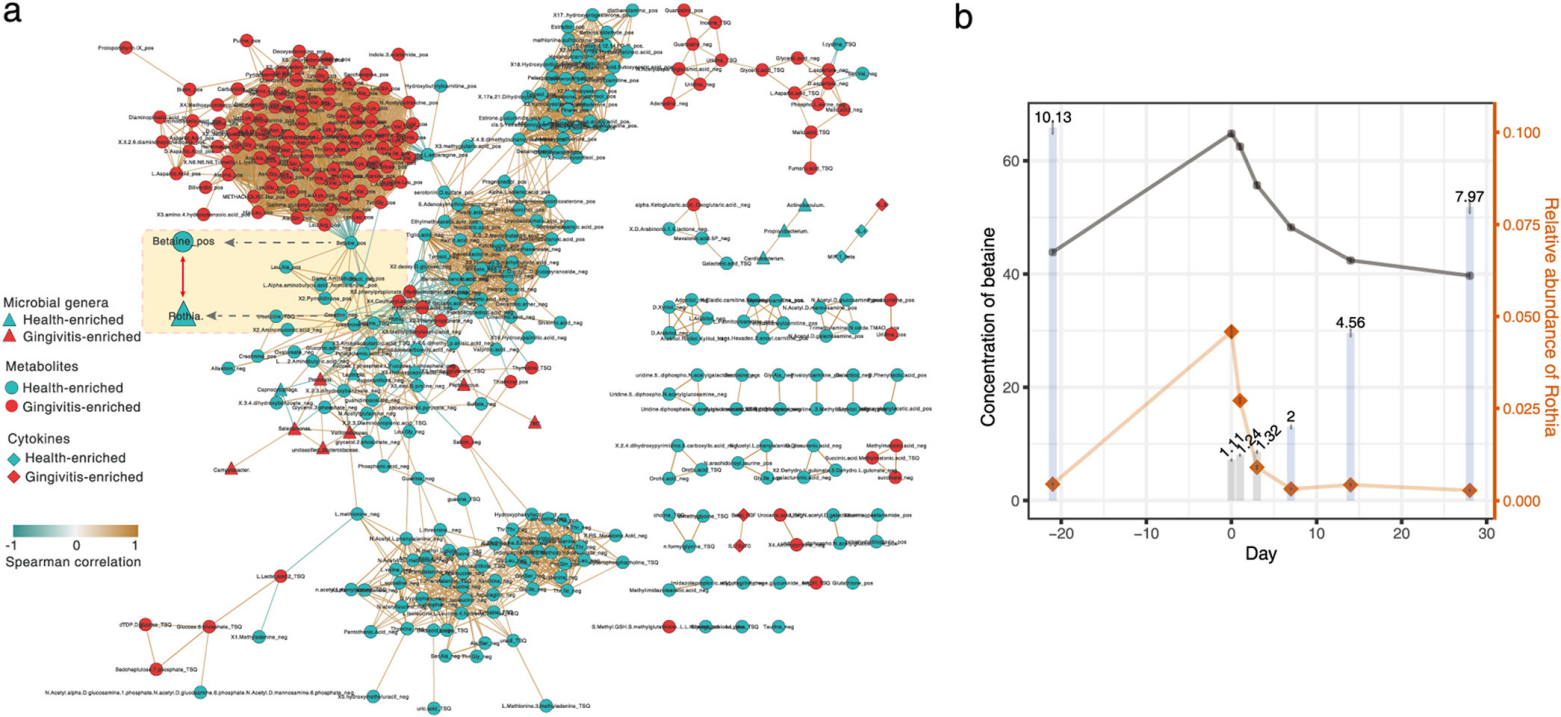
The interplay of plaque taxa, plaque metabolites, and salivary cytokines during gingivitis retrogression, onset, and progression. (a) Network analysis of microbial taxa and metabolites in the temporal program consisting of NG-baseline-EG. Negative correlations are shown in green, positive correlations are in blue, and predictive taxa are in gray. Edge weights represent the strength of correlation. *Rothia* and betaine have the largest number of connections (i.e., they are the hub nodes) and are highly correlated with each other. For the metabolite nodes, “pos” and “neg” indicate acquisition by positive and negative modes in the nontargeted metabolomic approach, while “TSQ” indicates acquisition from the targeted metabolomic approach. (b) Temporal covariation of betaine and *Rothia* during gingivitis retrogression and induction. The bar plot indicates the clinical symptoms (i.e., mean bleeding) at each of the time points (days −21, 0, 1, 3, 7, 14, and 28). The colors of the bars show statistical significance of bleeding between a given time point and baseline (day 0): blue, significant; gray, not significant.

We then built a co-occurrence network from the multi-omics data for biomarker discovery, by calculating the correlation matrix of all features via Spearman’s correlation analysis. The resulting network contained 27,942 total significant edges (|rho| > 0.6, false discovery rate [FDR] *P < *0.05) and 1,196 nodes that span features from all three types of measurement. A filtered subnetwork was further built from 29 bacterial genera, 304 metabolites, and 8 salivary cytokines that were differentially abundant between day 0 and 28 (Fig. 3a). Between-metabolite associations accounted for the vast majority (over 99%) of edges, clearly revealing complex and strong association among metabolites. In addition, 51 strong associations between microbial genera and metabolites were found, highlighting the impact of gingivitis onset and progression on microbe-dependent metabolisms in plaque. Among these, the *Rothia*-betaine link is one of the most prominent features in the network (red arrows in Fig. 3a). As a bacterial marker that is depleted in gingivitis, *Rothia* had the most links to metabolites (*n* = 14) and exhibited the strongest association with the metabolite of betaine (i.e., trimethylglycine [TMG]; rho = 0.7) (Fig. 3a), which is also depleted in gingivitis. In fact, the abundance of betaine and that of *Rothia* were highly synergic along the full 49-day course (Fig. 3b); moreover, both were negatively correlated with symptomatic severity of gingivitis; i.e., they were depleted from NG to baseline and then enriched again from baseline to EG, with the peaking of betaine and *Rothia* being coincident with the maximal healthy state of gingivae at baseline (Fig. 3b). Notably, the depletion rates of betaine and *Rothia* during EG induction are not constant: they both steeply decreased during the SoH stage and then gradually stabilized (Fig. 3b); in particular, at day 3, levels of *Rothia* dropped to 21% of the peak at day 0, bottomed out at day 7, and stayed so for the remaining 21 days. These observations suggest that the SoH stage, despite the lack of clinically observable changes in bleeding (versus baseline), is the most active and consequential phase in both microbiome structural change and the gingivitis-driving microbial metabolism.

Coincidentally, in addition to its synergy with bacteria that are more abundant in healthy gingivae, such as *Rothia*, betaine is negatively linked to many that are enriched in the presence of gingivitis, such as *Peptostreptococcus*, *Prevotella*, and *Treponema*, etc. (Fig. 3a). This suggests an important, perhaps protective, role of betaine in gingival inflammation. Accumulating evidence has shown that betaine plays an anti-inflammatory role in multiple inflammatory diseases, potentially by balancing hyperosmosis and protecting cells from shrinkage and death (28). Similarly, the positive link to betaine and the negative association with gingivitis severity indicate that *Rothia* is perhaps beneficial to gingival health and potentially contributes to betaine metabolism in plaque.

On the other hand, only three of the 27 cytokines tested are present in the network (Fig. 3a). MIP-1β is enriched in healthy gingivae, yet IL-9 is enriched in gingivitis and negatively correlated with MIP-1β (Fig. 3a): in fact, IL-9 is significantly downregulated at day 3 and day 7 and upregulated at day 28 (versus day 0; Fig. 2d). However, no specific associations between salivary cytokines and plaque taxa or plaque metabolites were found during the process of EG induction (Fig. 3a).

### Identifying microbiome links between gingivitis-SoH and periodontitis via meta-analysis.

**FIG 4:**
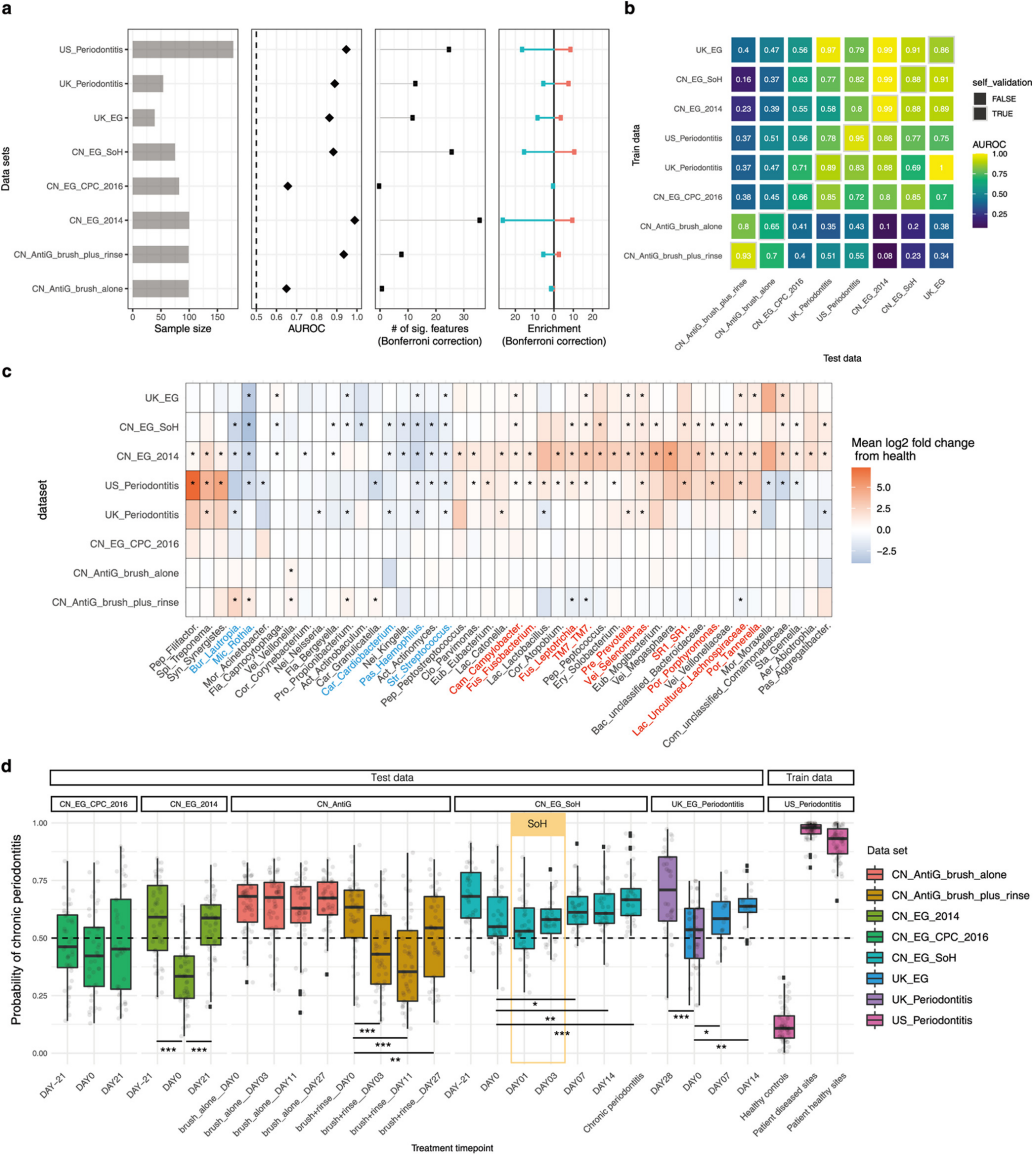
Meta-analysis of existing gingival microbiome data sets revealed similar microbial signatures between gingivitis SoH and periodontitis. (a) Most periodontal disease progression or retrogression shows microbiome alterations, with consistent disease-associated shifts that differ in their extent and direction. Panels show (from left to right) sample size for each study, area under the ROC curve (AUROC) for the genus-level random forest classifiers (*x* axis starts at 0.5, the expected value for a classifier that assigns labels randomly, and AUROC of <0.5 are not shown), number of genera with *q* values of <0.05 (Wilcoxon rank-sum test, Bonferroni correction) for each data set (if a study reveals no significant associations, no points are shown), and direction of the shifts in microbiome structure, i.e., the percentage of associated genera that are enriched in disease. (b) Cross-prediction matrix reporting prediction performance as AUROC values obtained using a random forest model on the genus-level relative abundance. Matrix values refer to the AUROC values obtained by training the classifier on the data set of corresponding row and then applying it to the data set of the corresponding column. The prediction accuracy between gingivitis and periodontitis is remarkably high, suggesting a strong microbial link between these two periodontal diseases. Moreover, the prediction accuracy between antigingivitis treatments is higher than that between EG experiments, suggesting that antigingivitis treatments often result in very similar microbiome responses, regardless of the difference in cohorts. (c) Heat map for log_2_ mean fold change of all plaque genera between the last day of treatments and baseline in each of the longitudinal studies (or between case and control groups in the cross-sectional studies). Blue denotes a reduction in relative abundances of genera (red indicates enrichment) versus baseline. Significant fold changes (Bonferroni-corrected *P < *0.05) are marked by asterisks, while nonsignificant changes (Bonferroni-corrected *P > *0.05) are indicated as blanks in the heatmap. The colors of the genus names indicate those showing highly consistent enrichment (red) or reduction (blue) in the periodontal disease state across data sets. (d) A random forests classifier of periodontitis was built based on the subgingival microbiomes in a U.S. periodontitis cohort and then applied to all the other data sets in the meta-analyses, so as to model the estimated probability of periodontitis for the gingivitis patients. FDR-corrected statistical significance: *, *P* ≤ 0.05.

To derive a microbiome-based view of the gingivitis-to-periodontitis transition (a process that can take decades), we conducted a meta-analysis of published microbiomes for gingival plaques, of sufficient sample size (>20 human adults) and with disease-associated (i.e., case or control labels) or time-revolved metadata (i.e., baseline or time point labels) (Table 1). Among the data sets found (all 16S rRNA amplicon based), six were publicly accessible; thus, collectively, 1,221 oral-microbiome samples were reanalyzed from raw sequences (via Parallel-META 3.0 [29] and the Oral Core microbiota database) (Table 1; Fig. 4a and b) for taxonomic profiles and metabolic functions (via PICRUSt [29, 30]) (Fig. S5b).

**TABLE 1:** Gingival-inflammation microbiome data sets used in the meta-analysis

Data set	Disease related	Sampling niche	Sampling method	Reference	Target region	Primers	Sequencing platform	DNA extraction kit	Sample size	Host population size	Host geolocation	Data source
CN_SoH	Gingivitis	Supragingival plaque	Plaques were collected with sterile Gracey curettes and then removed from the curettes with a cotton-tipped swab.	This study	V1-V3	5F–534R	MiSeq	QIAamp DNA minikit	261	40	Beijing, China	http://mse.ac.cn/SoH.html
CN_EG_ 2014	Gingivitis	Supragingival plaque	Supragingival plaque samples (along the gingival line within a 2-mm depth) from two entire quadrants (1 and 3 or 2 and 4) were collected by sterile Gracey curette at each visit.	13	V1-3	5F–534R	454	Bead-beating and lytic-enzyme-cocktail master mix were used for bacterial lysis; DNeasy blood and tissue minikits were also used.	150	50	Beijing, China	SRP022235, SRP022233
CN_EG_CPC_2016	Gingivitis	Supragingival plaque	Same as above	17	V1-3	5F–534R	454	Same as above	123	41	Beijing, China	SRP022233
CN_AntiG	Gingivitis	Supragingival plaque	Same as above	16	V1-3	5F–534R	454	Same as above	398	99	Beijing, China	SRP045295
UK_EG+Periodontitis	Gingivitis, periodontitis	Supragingival and subgingival plaque	Supragingival plaques were collected using a sterile curette from all the mandibular teeth with the exception of the third molars. Subgingival plaques were collected by inserting a curette to the full depth of pockets of >6 mm, after the removal of supragingival plaque.	15	V1-3	27F-519R	454	GenElute bacterial DNA extraction kit (Sigma-Aldrich)	92	20 + 20	London, UK	SRP026653
US_Periodontitis	Periodontitis	Subgingival plaque	After removal of supragingival plaque and drying of the target sites, subgingival samples were collected by insertion of four medium paper points for 10 s into three sites. Deep and shallow sites were sampled separately from subjects with periodontitis.	23	V1-2; V4	27F-342R	454	QIAamp DNA minikits	87 = 29(periodontitis shallow) +29 (periodontitis deep pocket)+ 29(Health)	58	USA	SRP009299
Meta-analysis	Healthy aging	Tongue/saliva	Participants were instructed to sample the surface of tongue with cotton swabs.	34	V4	515F-806R	Illumina	PowerSoil DNA isolation kit (most studies)	2003	216	U.S., Tanzania, Puerto Rico, etc.	EBI accession no. PRJEB5726, PRJEB5727, PRJEB5728, ERP016472, ERP012216, ERP016621, PRJEB6518, ERP008799, ERP008694, ERP021896

10.1128/mBio.03281-20.5 FIG S5 Conservation in microbiome functional change associated with the development of periodontal disease. (a) Heat map for the mean log_2_ fold changes of microbial metabolic functional pathways (with significance threshold Bonferroni-corrected *P < *0.05) in the plaque during the onset and progression of gingivitis, respectively. Bonferroni-corrected statistical significance: *, *P* ≤ 0.05. The absence of an asterisk indicates no significant change. The box plots indicate the individualized distances based on taxonomic compositions (species level) (b) or predicted functional pathways (c) between pairs of sampling groups related to disease development. Download FIG S5, PDF file, 2.6 MB. Copyright © 2021 Huang et al. 2021 Huang et al. https://creativecommons.org/licenses/by/4.0/ This content is distributed under the terms of the Creative Commons Attribution 4.0 International license.

We first tested whether the reported microbiome associations with the oral-disease states or the anti-gingivitis treatments can be recapitulated (Table 1). To compare such disease responses of microbiomes across studies, we first grouped all data into 10 data sets. Each data set could include samples from case and control groups in a cross-sectional study (e.g., UK_Periodontitis) or samples at the baseline and subsequent time points in a longitudinal study of EG (such as CN_EG_2014) or an anti-gingivitis treatment (such as CN_AntiG_brush_plus_rinse). Next, for each data set, we built a genus-level RF classifier to distinguish disease states (gingivitis or periodontitis) from the health states longitudinally or cross-sectionally and then compared their AUROC across data sets.

Surprisingly, periodontal disease status can be classified between hosts or within hosts (AUROC > 0.7) in all studies (Fig. 4a). Notably, the states of gingivitis and chronic periodontitis are highly distinguishable by plaque microbiome (AUROC > 0.9) in six of eight related data sets (Fig. 4a). We then asked whether and to what extent the microbiome-based RF classifiers of periodontal disease states can be applicable from one data set to another (Fig. 4b). For gingivitis, we observed very limited degradation in prediction accuracy for the cross-trained RF models from one cohort to another (AUROC ranges from 0.88 to 0.99 in either self-validation or prediction). Moreover, an RF classifier trained on periodontitis can be readily applicable to gingivitis or vice versa (AUROC > 0.75 in either self-validation or prediction), despite the large technical difference (or other non-disease-related biological differences) between studies/cohorts in the microbiome data that frequently confound such cross-applications (Fig. S4a). Thus, the gingivitis and periodontitis classifiers share a large number of microbial markers, suggesting a high degree of similarity in the underlying microbiome.

10.1128/mBio.03281-20.4 FIG S4 Generalizability of periodontal disease models revealed by cross-application of random forests models. (a) PCoA of all plaque microbiomes in the meta-analysis. Each dot represents a plaque profile at the genus level and is colored according to study ID. (b) The majority of disease-associated microbiome associations overlap a nonspecific microbial response to the periodontal disease. The bar plot indicates nonspecific and disease-associated genera (green), dataset-specific disease-associated genera (red), and nonmarkers (grey) found in all datasets. Nonspecific genera are associated with health (or disease) in at least two datasets. Dataset-specific genera are associated with health (or disease) in only one study or are associated with health (or disease) in the different direction for all the other studies. (c and d) Abundance and prevalence of nonspecific genera in all microbiomes in all data sets. Nonspecific genera on the *x* axis are as defined above. Download FIG S4, TIF file, 2.1 MB. Copyright © 2021 Huang et al. 2021 Huang et al. https://creativecommons.org/licenses/by/4.0/ This content is distributed under the terms of the Creative Commons Attribution 4.0 International license.

Next, the microbial signatures associated with gingivitis or periodontitis were compared across these data sets (see Materials and Methods). First, we asked whether the identified microbial response to gingivitis onset (i.e., SoH) or progression is consistent with reported gingivitis microbiome in these independent cohorts. Here, 1,023 samples (931 from China and 92 from the United Kingdom) from five gingivitis-related data sets were compared, each with a longitudinal design that tracks microbiome dynamics along gingivitis progression or retrogression. For cross-study comparison of microbial responses, statistical analyses on samples from the baseline and the last time point in each study were performed (with univariate tests on genus-level CLR-transformed relative abundances conducted for each data set independently, and the results were compared across studies (Wilcoxon rank-sum test with the Bonferroni correction). Notably, the gingivitis-associated microbiomes are highly reproducible across studies (Fig. 4c). In the EG data sets, microbiome shifts are characterized by enrichment of a large proportion of “pathogenic” or pathogen-associated genera and depletion of a few commensal oral bacteria (consistent across studies) (Fig. 4c). The EG-associated microbiome identified from our previous study (i.e., CN_EG_2014) harbored the broadest spectrum of microbial shifts (*n* = 41), among which >60% of microbial markers (e.g., *Rothia*, *Haemophilus*, *Actinomyces*, *Streptococcus*, *Selenomonas*, *Prevotella*, *Leptotrichia*, uncultured *Lachnospiraceae*, and TM7) actually overlapped those identified in other gingivitis progression studies (including the present gum SoH study) (Fig. 4c). Moreover, the two antigingivitis treatments of brushing alone and brushing plus rinsing (16)) are both characterized by enrichment of health-associated bacteria and depletion of pathogenic bacteria; in fact, the microbial taxa shifted toward the healthy state during gingivitis retrogression and largely overlapped markers of the EG studies (e.g., *Lautropia*, *Rothia*, *Granulicatella*, TM7, and *Leptotrichia*) (Fig. 4c) but in the opposite direction of abundance change.

Second, we tested whether or to what extent the stage-specific plaques of gingivitis are linked to those of periodontitis. Specifically, 260 samples were collected from two case-control studies (United Kingdom, *n* = 92; United States, *n* = 178) on periodontitis microbiomes: the UK_Periodontitis data set was from a study in which Kistler et al. profiled plaque microbiome of chronic periodontitis (15), and the US_Periodontitis data set was from a study in which Griffen et al. compared subgingival plaque microbiota from 29 periodontally healthy controls and 29 subjects with chronic periodontitis (including periodontally healthy and diseased sites) from a U.S. cohort (23). Notably, the periodontitis microbiomes feature a large number of genera that overlap those identified in the EG or even the SoH stage of gingivitis (Fig. 4b and c; Fig. S4). The microbiome shifts responding to chronic periodontitis in the U.S. or UK cohorts were characterized by an enrichment of gingivitis-enriched genera (such as *Porphyromonas*, *Leptotrichia*, *Selenomonas*, TM7, *Prevotella*, uncultured *Lachnospiraceae*, *Campylobacter*, *Fusobacterium*, and *Tannerella*) and a depletion of gingivitis-depleted ones (such as *Rothia*, *Haemophilus*, *Actinomyces*, *Streptococcus*, and *Kingella*). Importantly, those gingivitis-associated microbes were all identified as such in the Chinese cohorts. Considering the potential heterogeneity between cohorts (i.e., different geographic locations) or technical interstudy batch effects (such as a 454 versus Illumina sequencing platform, different primer sets, etc.), the very limited variation in microbial response to periodontal diseases across the two UK/U.S. periodontitis cohorts and the China gingivitis cohort is remarkable.

To validate the similarity in microbiome signature between gingivitis and periodontitis, we built an RF classifier of the chronic periodontitis on the plaque microbiome and applied this model to a given sample from any of the gingivitis stages for estimating its microbiome-based probability of periodontitis (which we designated the microbiome-based periodontitis index [MPI]) (Fig. 4d). In the training data set (i.e., US_Periodontitis), MPIs of the healthy controls are on average only 10%, while they average as high as 99% in periodontitis patients. In our present study, MPIs increased progressively along the EG process, a pattern that is consistent with the other EG data sets. In particular, the MPI at day 7 (end of the gum SoH stage), with a median of ∼62%, is significantly higher than that at day 0, suggesting the emergence of a periodontitis-like microbiome at this stage, due to the aforementioned profound changes in plaque microbiome, plaque metabolome, and host immunity that take place at the SoH stage.

### Temporal dynamics of microbiome in the development of gingivitis and periodontitis.

**FIG 5:**
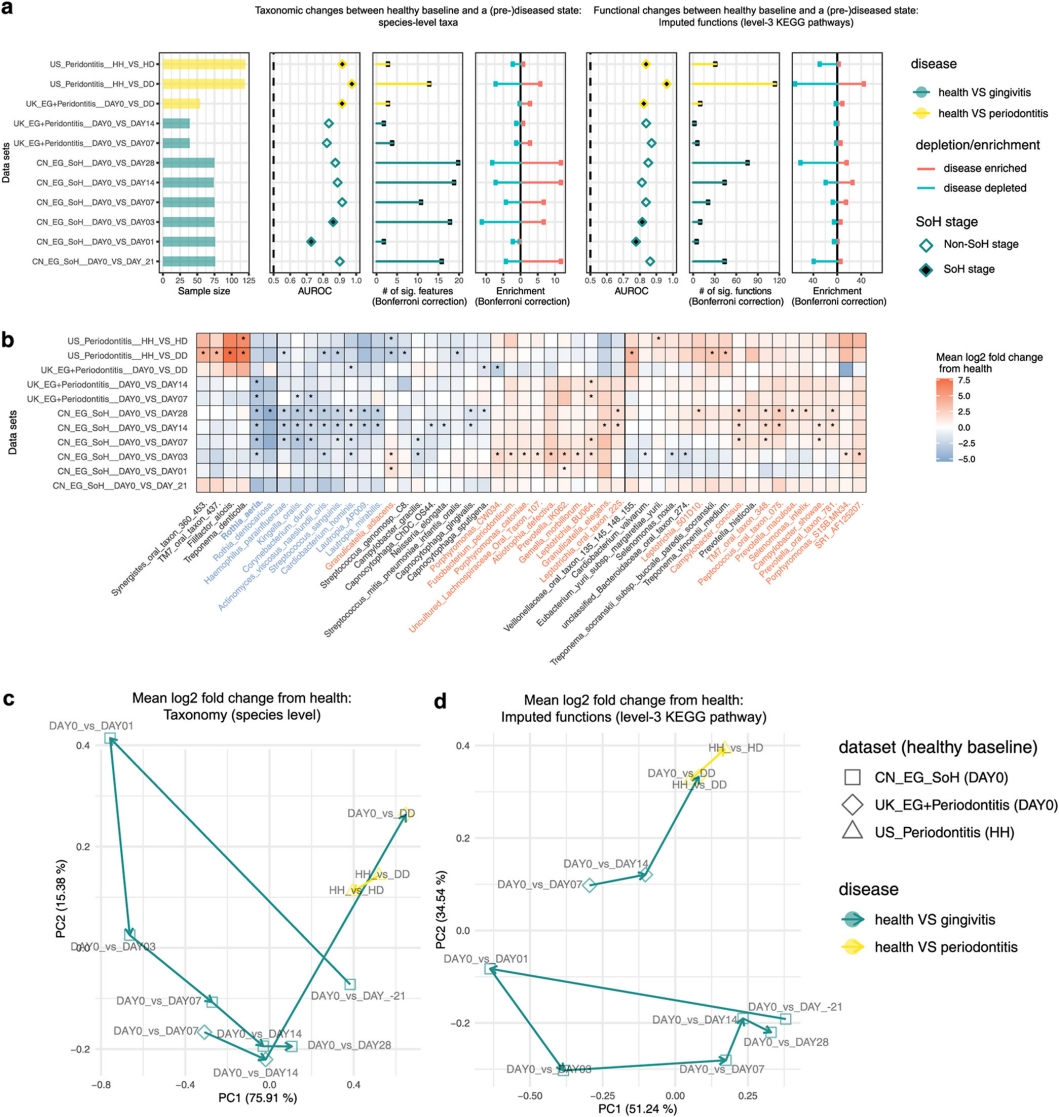
Comparing temporal microbiome shifts along the stages of periodontal disease. (a) Most oral-disease progression shows microbiome alterations, with consistent disease-associated shifts that differ in their extent and direction. Panels (left to right) show sample size for each study, area under the ROC curve (AUROC) for the species-level RF classifiers (the *x* axis starts at 0.5, the expected value for a classifier that assigns labels randomly; those with AUROC of <0.5 are not shown), number of species with *q* values of <0.05 (Wilcoxon rank-sum test, Bonferroni correction) for each data set, and direction of the shifts in microbiome structure, i.e., percentage of associated species that are enriched in the disease state; the last three panels show results of similar analyses conducted on the imputed functional profiles from 16S rRNA sequencing data. (b) Heat map for log_2_ mean fold change of bacterial species between a (pre)diseased state and the healthy baseline in each. Blue denotes reduction in relative abundances of species (red indicates enrichment) versus baseline. Significant fold changes (Bonferroni-corrected *P < *0.05) are marked by asterisks, while nonsignificant fold changes (Bonferroni-corrected *P > *0.05) are blank in the heat map. We next performed PCoA based on the mean log_2_ fold change data of species (c) or predicted functional pathways (d) that are associated with two oral diseases. Each dot in the PCoA plots represents a process of microbiome alterations from health to the onset or progression stage of a given oral disease. The dots are colored by disease. The lines with arrows represent the path along which microbial alterations occurred during disease development.

To characterize the temporal shifts in both species-level taxonomic diversity and functional diversity during periodontal disease development, we performed a meta-analysis of the published SoH, UK_EG, UK_Periodontitis, and US_Periodontitis data sets (Table 1). We classified the disease status (or time points) using RF models based on either the species-level taxonomic profile or the predicted functional profile (by PICRUSt) through the stages of disease development in all studies (Fig. 5a). Surprisingly, AUROC of species-level-taxonomy based RF classifiers for plaque at day 3 reached 0.85 (function-based classifiers, 0.81), which is already quite close to the value of 0.88 at day 28 (function-based classifiers, 0.85). Thus, plaque functional profiles already resemble that of the severe gingivitis stage within 24 h after dental scaling (Fig. 5a) and actually saturate after 24 h. The discriminative power of this function-based classifier (AUROC = 0.78) is nearly equivalent to that distinguishing chronic periodontitis patients from healthy individuals from the UK cohort (AUROC = 0.82; DAY0_VS_DD), suggesting an ultrarapid assemblage of functional components in the plaque biofilm that closely resemble those in periodontitis patients.

Moreover, to test whether microbiome successions are concordant between the developmental stages of these oral chronic inflammations, we quantitatively compared the microbial differential abundance profiles between time points or disease severities. For each data set, the differential abundance (i.e., mean log_2_ fold change) of microbial features in the plaque microbiome from healthy baseline to a given developmental stage of disease was measured (Fig. 5a and b; Fig. S5a). For two given microbial signatures (e.g., day 0 versus day −21 and day 0 versus day 28 in the SoH study), we first ranked the features by the degree of differential abundances in each of them and then calculated the Pearson’s correlation between these two feature ranking lists. To reveal the patterns driving the temporal difference in microbiome across diseases, we next performed PCoA via the correlation-based distance metric of all pairs of feature ranking lists, with each dot in PCoA corresponding to a pattern of microbial alteration between the healthy baseline and a particular disease developmental stage (instead of a microbiome sample) (Fig. 5c and d).

Intriguingly, at the species level, the microbiome differences during gingivitis development are more pronounced than those from periodontitis (Fig. 5c). During gingivitis progression, along PC1, the profile of microbiome alteration between the baseline (day 0) and a given time point increasingly resembled that between health and periodontitis in either the U.S. or the UK cohort. Notably, the microbial taxonomical response to severe gingivitis (e.g., day 0 versus day −21 and day 0 versus day 28 in the SoH study) is highly similar to that of chronic periodontitis. Thus, taxonomic perturbations during dysbiosis are highly consistent between gingivitis and chronic periodontitis (Fig. 5c). Notably, during gingivitis development, functional potential of microbiome is relatively conservative over time, particularly after the SoH stage (Fig. 5d). In fact, the gingivitis-associated community in dental plaque biofilm actually assembles rather rapidly in the very early stage (i.e., the SoH stage), to form a “climax”-like community configuration that is very similar to the periodontitis-associated community (Fig. 5d).

### Gingivitis onset and development greatly accelerate oral microbiome aging.

“Healthy” aging in humans is accompanied by increased incidence of periodontal disease (31, 32). We previously showed that, in 4- to 6-year-old children, the supragingival plague microbiome, which defines a personalized oral microbiome age (OMA), is correlated with human chronological age in heathy (i.e., caries-free) children (33) but deviates from the latter upon caries onset; moreover, we found that, for healthy adults 18 to 63 years old, the oral microbiome can predict human chronological age with a mean absolute error of 2.44 years (34). These observations revealed and underscore the significance of a healthy oral-microbiome-aging process.

**FIG 6:**
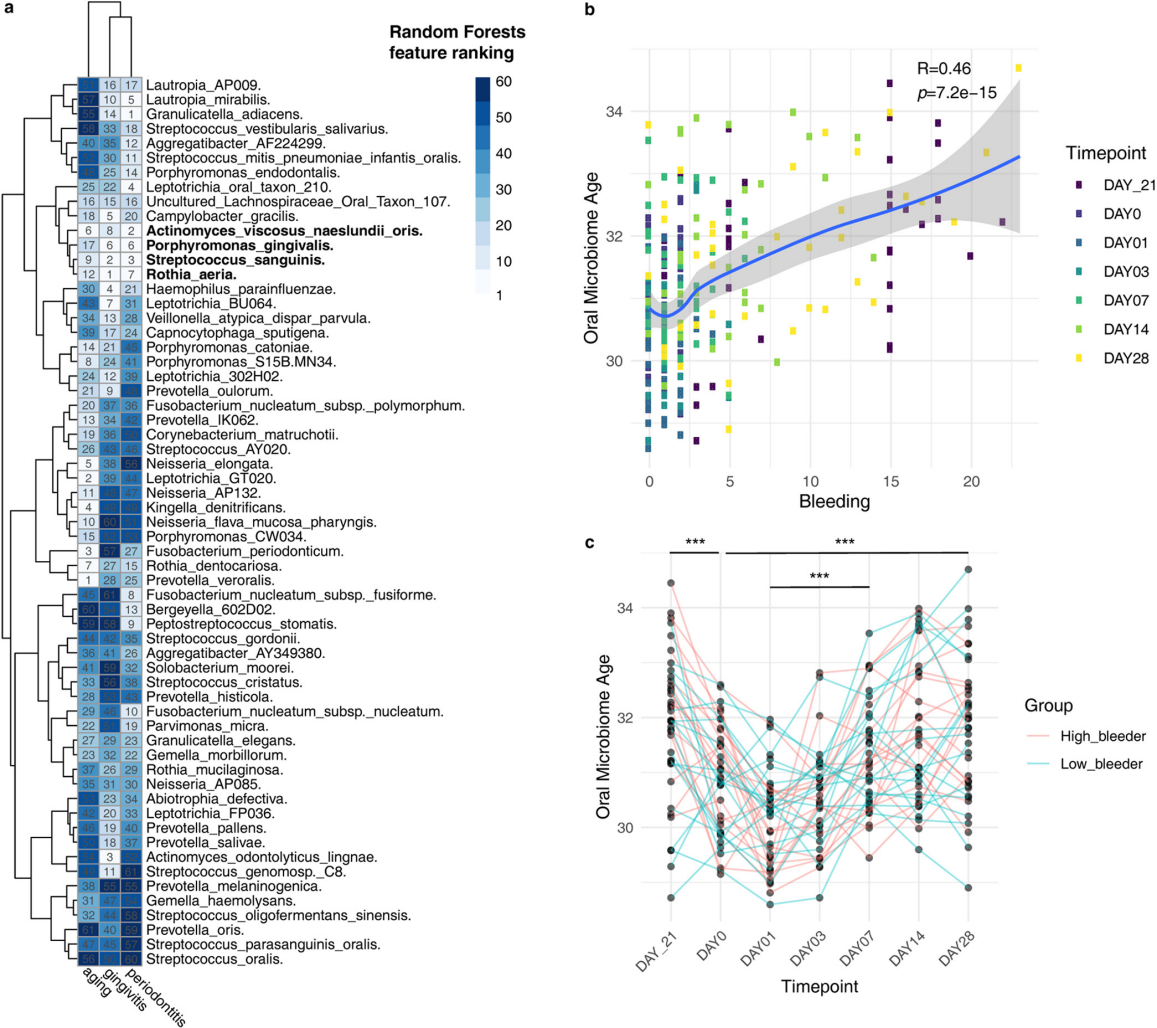
Gingivitis development greatly accelerates oral microbiome aging. (a) The ranking relevance of each species in the random forest models for predicting oral aging, gingivitis, and periodontitis and identification of a minimal microbial signature for them. Only species (*n* = 61) appearing in all data sets are reported. (b) The scatterplot shows the correlation between oral microbiota age predicted and bleeding in the SoH study. (c) The linked dot plot shows the temporal dynamic of oral microbiota age (OMA) within participants in the experimental gingivitis study. FDR-corrected statistical significance: *, *P* ≤ 0.05; **, *P* ≤ 0.01; ***, *P* ≤ 0.001.

To test whether and how such a process is linked to the temporal change of the microbiome from healthy gum to gingivitis and then to periodontitis, we benchmarked the microbial signatures associated with gingivitis, periodontitis, and healthy host aging. The membership and enrichment directionality of key microbial features involved in these phenotypic changes were systematically investigated. We respectively trained the RF models for predicting healthy aging (regression), gingivitis (classification) in the SoH study, and periodontitis (classification) in the US_Periodontitis study using the abundance profiles of shared species across the three data sets. Then we ranked the microbial features by their importance estimated by built-in RF importance score and compared the ranking relevance across machine learning models (Fig. 6a). Comparison of the ranking lists revealed related yet distinct roles that each oral bacterium plays in healthy mouth aging, experimental gingivitis, and chronic periodontitis (Fig. 6a). Intriguingly, a number of features emerged that consistently showed high importance ranking in predicting the three processes (Fig. 6a): e.g., Streptococcus sanguinis, Rothia dentocariosa, and Rothia aeria are depleted with healthy host aging and with gingivitis and periodontitis; in contrast, Porphyromonas gingivalis increased with aging and was also enriched in patients with severe gingivitis and periodontitis.

Next, we contextualized the gingivitis-associated microbiome change, which takes place within a short time frame, with oral microbiome changes in the decades-long aging process of human adults. Given the intrinsic technical effect between studies, we retained 61 commonly shared features in the training and test data sets for model application. These 61 species can account for 54% of features in the training data and 40% of species in the test data. We retrained the oral microbiome aging model using these 61-species abundance profiles. The prediction accuracy of this reduced model (mean absolute error [MAE] = 2.44 years) can still achieve an accuracy level similar to that using the full set of microbial species, suggesting that the most important features have been retained. OMA was then calculated for each of the subjects in the longitudinal study design. Interestingly, OMA is strongly correlated with the number of sites of bleeding, which is the main symptomatic measure of gingivitis (*r *= 0.46, *P < *1e−14). In fact, every 10-bleeding-site increment would result in at least 1 additional year of oral microbiome age (Fig. 6b).

Furthermore, projecting OMA onto this NG-baseline-EG data set suggested that the metric reliably reflects the temporal dynamics of gingivitis symptoms within hosts over time (Fig. 6c). For example, in the NG phase and at day 28 of EG, every subject’s OMA peaked; however, dental scaling dramatically reduced OMA to the lowest level at day 0 and 1. In particular, the cessation of oral hygiene elevated OMA by one year on average over the 28-day experiment, i.e., an acceleration of oral microbiome aging by at least 1 order of magnitude of chronological aging. Such poor-hygiene-induced acceleration of oral microbiome during its already fast aging highlights the importance of oral hygiene in maintaining healthy aging of the oral ecosystem.

## DISCUSSION

Despite the technological challenges, integrating the human dental plaque microbiota and metabolomics profiles enables an in-depth and mechanistic understanding of periodontal disease etiology. Simultaneous analysis of dental plaque samples via DNA sequencing and LC-MS/MS has been hindered by (i) the low biomass of dental plaque sampled with high temporal resolution from each host and (ii) the difficulty of reconciling the distinct sample preprocessing procedures for DNA sequencing and LC-MS/MS on a plaque sample (e.g., the organic solvent extraction in LC-MS/MS can reduce the DNA quality for sequencing). Therefore, in our new strategy, two dental plaque samples (up to 14 teeth each) were collected (for each subject) from quadrants 1 and 3 (plaque A) or 2 and 4 (plaque B) for sequencing and LC-MS/MS, respectively (randomly assigned, to eliminate potential bias). This is particularly enabling for recording the integrated metagenome-metabolome choreography of plaque when sampled at high temporal resolution and particularly during the SoH phase (just 0 to ∼3 days away from baseline, with especially low plaque biomass).

The link and distinction temporal dynamics among host symptoms, immune factors, plaque structure, and plaque metabolome unveiled how plaque the microbiota drives gingivitis onset and progression. Most importantly, an asymptomatic SoH state of gingivae, from 0 to 3 days after dental prophylaxis and pause of oral hygiene, was uncovered, when the most intense host-microbiome interactions take place, i.e., rapid and consistent alterations in plaque microbiota, metabolite pool, and salivary cytokines. In particular, during this preclinical-symptom, very transient gingival state of SoH, plaque residents (e.g., *Rothia* spp.) and metabolites (e.g., betaine) that are strongly negatively correlated with gum bleeding (over the entire 49-day NG-baseline-EG process) undergo a steep decrease, while at least 11 salivary cytokines dramatically change in response (six upregulated and five downregulated compared to day 0) and then rapidly plateau. In contrast, such alterations were not seen in subsequent phases of gingivitis development (e.g., from day 7 to 28), even for those with much higher symptomatic severity.

Betaine was not previously linked to gingivitis development, despite its being recognized as maintaining cell osmotic pressure, which can promote cell survival under the high hyperosmotic pressure potentially due to inflammation and diseases (28). Interestingly, it is at present an ingredient in toothpaste for relieving dry mouth (35). In our plaque samples, betaine consistently and continuously declined as the gingivitis developed (particularly in the SoH stage), suggesting a protective role against gum inflammation. Notably, its concentration in the plaque was highly correlated with plaque residents, such as *Rothia* spp., that were enriched in healthy gums and depleted in patients with gingivitis. Therefore, the health-associated members of plaque might have served as a source of betaine that possibly to protect the gum from gingivitis, which underscores the importance of maintaining a healthy plaque.

Notably, although taxonomic shift in plaque took place as early as 24 h after dental prophylaxis (by acquiring microbial colonizers from saliva [11, 20]), it was accompanied by a delayed functional shift, as revealed by plaque metabolome analysis. This suggests that establishment of primary colonists in plaque alters the plaque metabolome within 48 h (i.e., at or by day 3), which then elicits both gingival inflammation and subsequent plaque development, starting a detrimental cycle: periodontal tissue destruction by plaque dysbiosis provides nutrients for bacterial growth, which further promotes dysbiosis and tissue inflammation (11). Therefore, despite its apparent baseline-like characteristics, the SoH phase is a transient yet crucial time window to prevent or abolish the start of such vicious circles.

In the co-occurrence network analysis, no association was observed between cytokines and the microbial taxa that become prevalent during EG. There are two potential explanations. First, temporal dynamics of microbiome features and cytokines were quite distinct throughout this EG experiment. The proinflammatory cytokines drastically changed only in the first 3 days and plateaued afterward, whereas most disease-associated plaque microbial features continuously shifted toward a periodontitis-like community configuration throughout the 28-day experiment. This result may imply that innate (inflammatory) immunity and acquired immunity are coordinated to modulate the homeostasis of periodontal tissue (36). In the initial stage of gingivitis (i.e., SoH), a group of cytokines act in the first wave of the inflammatory response to dental plaque accumulation and stimulation. If such short-term periodontal inflammation is not resolved, chronic pathology may occur, where the adaptive immune response is activated. At this stage, hosts can tolerate the plaque microbiome, i.e., the inactivation of those cytokines. Second, in this study, we collected salivary samples for cytokine measurement rather than the gingival crevicular fluid (due to the technical challenges associated with the latter). This can also result in the apparent lack of associations between salivary cytokines and the EG-associated microbes.

Surprisingly, implication of this SoH stage is supported by our meta-analysis of past oral-microbiome studies, which reveals a microbiome-mediated link between the very early stage (i.e., SoH of gingivitis) and very late stage (periodontitis) of periodontal disease. Gingivitis and periodontitis patients share a significant number of bacteria genera (18–20, 23), and periodontal treatments can result in depletion of disease-associated bacteria and enrichment of health-associated ones in plaque (16, 17, 37). However, systematically tracking microbial associations across different stages for chronic periodontal diseases remains a challenge, since it is impractical to create or modulate advanced disease states directly in humans, while clinical studies can only induce mild or moderate disease states (notably, this holds true for many chronic diseases). Moreover, technical variations such as interstudy differences in the sequencing protocol, 16S databases, or statistical methods prevent comparison of microbial associations across studies (38). For example, microbiome data are compositional (39); however, in many past studies, traditional statistical methods such as the *t* test or Wilcoxon rank-sum test were widely and inappropriately used on the raw abundance data for microbial marker discovery; in fact, once the compositionality issues in statistical analysis are accounted for, it is far less clear whether the reported microbial associations can be recapitulated (39).

To tackle these issues, we reanalyzed from raw data all published and accessible microbiome data sets with consistent parameters and RF models. Our results profoundly relate gingivitis to periodontitis via the plaque microbiome. Specifically, (i) the oral-microbiome responses to a disease state, either gingivitis or periodontitis, can be highly consistent across human populations, while this is not the case for most of the other chronic diseases (33, 38), and (ii) the plaque residents specifically responding to periodontal inflammation are quite consistent between the very early stage of gingivitis (i.e., SoH) and the eventually irreversible and detrimental state of periodontitis, despite their decades-long temporal gap and the large host- or technology-related variation among cohorts/studies. This is in contrast to early childhood caries (ECC), where plaque microbiomes at the new-onset stage are very distinct from those at the late stage (33, 40). The patterns and nature of such microbiome changes underlying chronic disease development, whether conserved or divergent among the many chronic inflammations at oral or other human body sites, can shed new light on disease etiology and help refine diagnosis, prevention, and treatment.

Intriguingly, the change of plaque microbiome within the 28-day baseline-to-EG segment resembles the oral microbiome shifts in the decades-long aging process of healthy adults. Such similarity is underpinned by (i) a progressive decline in the immune functions during normal aging, exacerbating the inflammatory responses to oral bacterial infection and potentially causing the pathogens residing the oral mucosa to proliferate; (ii) the aging-related changes in the host immunity, which drastically modified the ecological niches of oral residents, so those enriched (or depleted) in periodontal disease (e.g., Porphyromonas gingivalis and *Rothia* spp.) became more (or less) abundant during normal aging. Therefore, EG can perhaps serve as a personalized, longitudinal research model for oral-ecosystem development and aging in humans (which would otherwise take decades to track). On the other hand, gingivitis development accelerates oral microbiota aging (OMA) by 10-fold, with both featuring dramatic depletion of *Rothia* spp. and elevation of levels of Porphyromonas gingivalis (brain colonization by which can result in amyloid plaques, a hallmark of Alzheimer’s disease [8]). This novel microbiome link between gingivitis onset/development and healthy aging of the oral ecosystem sheds new light on the significance of daily oral hygiene and raises intriguing hypotheses on maintaining or reversing healthy oral-ecosystem aging by targeted manipulation of gingivitis-induced oral-microbiota members or metabolites (such as betaine).

In summary, by tracking the choreography of plaque microbiome structure, plaque metabolome, and host immune response during gingivitis onset and progression, we unraveled a microbiome-defined SoH stage of gingivitis, i.e., the 24 to 72 h after a pause in oral hygiene. Although transient and asymptomatic, SoH is a crucial phase when the most intensive changes in plaque structure and metabolism as well as host immune factors take place, and it exhibits a microbial signature highly similar to that of periodontitis. Prevention or treatment of SoH would eliminate the risk of dramatically accelerated oral microbiome aging by avoiding full gingivitis development. In light of the epidemic of periodontal disease (1–5) and the insufficient public health awareness of the importance of oral hygiene (a significant portion of the world’s population still fails to brush teeth daily [3, 4]), our findings underscore the importance of intervening at the SoH stage of gingivitis via proper oral-hygiene practices, so as to maintain a healthy, periodontitis-preventive plaque, plus a healthy aging process of the oral ecosystem. On the other hand, the host- or microbiome-associated factors that influence key features of such a gingival SoH stage (e.g., its timing, duration, etc.) are not yet clear, and it would be intriguing to probe whether diet (or lifestyle changes of the human race over the past few centuries) may play a role. Finally, since SoH appears to be a shared stage that carries disease-specific microbial, metabolomic, and immunological features, defining and comparing the SoH states of various types of chronic polymicrobial inflammations will be of great interest and should introduce new opportunities for predictive and personalized medicine.

## MATERIALS AND METHODS

### Overall design of the study.

The notion of experimental gingivitis was established as a noninvasive model in humans for the pathogenesis of gingivitis (13). This single-center, examiner-blind, controlled clinical trial was conducted at the Procter & Gamble (Beijing) Technology Co., Ltd., Oral Care Department, with approval from the P&G Beijing Technical Center (China) institutional review board and in accordance with the World Medical Association Declaration of Helsinki (1996 amendment). ICH guidelines for good clinical practice were followed. All participants gave written informed consent prior to the study.

### Overview of the human cohort.

A total of 40 volunteers who met all inclusion criteria participated in this study, and all completed it (Table S2). Clinical examination of gingival tissues using the Mazza index was conducted at all of the visits by a qualified dental examiner (Fig. 1a). For each subject, supragingival plaque and salivary samples were collected by professional dentists at day −21 (NG), day 0 (baseline), day 1 (EG), day 3 (EG), day 7 (EG), day 14 (EG), and day 28 (EG), in a longitudinal manner (Fig. 1a). The optimal gingival health state on day 0 was achieved through dental prophylaxis and rigorous oral hygiene during the oral-hygiene phase prior to baseline. Dental prophylaxis, including supra- and subgingival whole-mouth cleaning on a total of 28 teeth, was performed on day −21, day −14, and day −7. Subjects were instructed to brush with a sodium fluoride dentifrice 3 min each time twice daily in the oral-hygiene phase. In contrast, in the EG phase (day 0 to day 28), only rinsing with purified water was allowed for each of the subjects.

10.1128/mBio.03281-20.7 TABLE S2 Overview of the 40-individual cohort that underwent the controlled oral-hygiene regimen. (a) Overview of the cohort. (b) Background information on each subject that participated in the cohort. Download Table S2, PDF file, 0.04 MB. Copyright © 2021 Huang et al. 2021 Huang et al. https://creativecommons.org/licenses/by/4.0/ This content is distributed under the terms of the Creative Commons Attribution 4.0 International license.

### Clinical assessment.

A qualified dental examiner performed oral-tissue assessments on the study participants at day −21, day −14, day −7, day 0, day 1, day 3, day 7, day 14, and day 28. Assessment of the oral soft tissue was conducted via a visual examination of the oral cavity and perioral area. The structures examined include the gingiva (free and attached), hard and soft palate, oropharynx/uvula, buccal mucosa, tongue, floor of the mouth, labial mucosa, mucobuccal/mucolabial folds, lips, and perioral area. Assessment of the oral hard tissues was conducted via a visual examination of the dentition and restorations. Gingivitis was assessed based on the Mazza index (13): sampling was performed on the mesiofacial and the distolingual part of each tooth, for a maximum of 56 sites.

### Saliva sample collection.

At the day −21, day 0, day 1, day 3, day 7, day 14, and day 28 visits, subjects were asked, prior to plaque sampling, to expectorate approximately 10 ml of unstimulated saliva into a labeled tube (Fig. 1a). The samples were frozen at −20°C immediately after collection until use for cytokine profiling.

### Plaque sample collection.

Gingival plaque from each of the 40 subjects was collected at day −21, day 0, day 1, day 3, day 7, day 14, and day 28 (Fig. 1a). Specifically, subjects refrained from oral-hygiene practices, including toothbrushing, flossing, or mouth rinsing, on the morning of sampling, and supragingival plaque samples along the gingival margin were collected after gingival index examination using a Gracey curette by a qualified dentist. At each time point, to ensure a sufficient amount of plaque for analysis, samples were taken from each subject’s maxillary right and mandibular left quadrants or maxillary left and mandibular right quadrants alternatively. All samples were stored at −70°C until use.

### Plaque microbiome structure analyses.

Genomic DNA was extracted from the plaques. Barcoded 16S rRNA amplicons (V1-V3 hypervariable region) of all the 261 samples were sequenced with an Illumina MiSeq system. All 16S rRNA raw sequences were preprocessed following the standard QIIME (v.1.9.1) pipeline (41). Downstream bioinformatics analysis was performed using Parallel-META 3 (29), a software package for comprehensive taxonomical and functional comparison of microbial communities. Clustering of operational taxonomic units (OTUs) was conducted at the 97% similarity level using the OralCore database (42). Taxonomically assigned sequences were further agglomerated at the genus level for structural comparison of microbiomes.

### LC-MS/MS data acquisition for plaque metabolome.

Prior to LC-MS/MS analysis, plaque samples were prepared using the following procedures. For extraction, 1 ml (40:40:20 [by volume]) methanol (MeOH)/acetonitrile (ACN)/water was added to the preweighted supragingival plaque in a 2-ml polypropylene (PP) tube and vortexed for 1 min. Plaque pallets in the extraction solvent were incubated in 95°C water bath for 1 h, centrifuged at 3,000 rpm, and subsequently transferred to another 2-ml PP tube. For complete extraction, 500 μl extraction solvent was added as described above to the original tube, vortexed for 10 s, and centrifuged at 3,000 rpm for 10 min. Each of the final extraction solutions was combined with the one obtained in the last step. Each liquid extraction was dried completely with nitrogen and then stored in a −80°C freezer until use.

Nontargeted metabolomic analysis was performed using a Q Exactive Orbitrap spectrophotometer (Thermo Fisher, CA). After resuspension of the dried extract, each of the samples (1 μl supernatant) was loaded onto a normal-phase chromatography column and then eluted to the Orbitrap mass spectrometer with an aqueous phase containing 5 mM ammonium acetate as the eluent from 1% to 99% within 15 min. The stationary phase was 95% acetonitrile with 5 mM ammonium acetate. Data with a mass (*m/z*) range from 100 to 1,500 was acquired in the positive ion mode using data-dependent MS/MS acquisition. The full scan and fragment spectra were collected with resolutions of 70,000 and 17,500, respectively. The source parameters are as follows: spray voltage, 3,000 V; capillary temperature, 320°C; heater temperature, 300°C; sheath gas flow rate, 35 arb.; auxiliary gas flow rate, 10 arb. Metabolite identification was based on Tracefinder search with a home-built database containing 529 compounds.

Targeted metabolomic experiments were performed on a TSQ Quantiva mass spectrometer (Thermo Fisher, CA). C_18_-based reverse-phase chromatography was utilized with 10 mM tributylamine–15 mM acetic acid in water (pH ∼6) and 100% methanol as mobile phases A and B, respectively. This analysis focused on tricarboxylic acid (TCA) cycle, glycolysis pathway, pentose phosphate pathway, amino acids, and purine metabolism. A 25-min gradient from 5% to 90% mobile phase B was used. Positive-negative ion switching was performed for data acquisition. Cycle time was set as 1 s, and a total of 138 ion pairs were included. The resolutions for Q1 and Q3 are both 0.7 FWHM (full width, half maximum). The source voltage was 3,500 V for positive and 2,500 V for negative ion mode. Sweep gas was turned on at 1 arb. flow rate.

### LC-MS/MS data analysis for plaque metabolome.

For targeted metabolomics, a triple-quadrupole mass spectrometer (TSQ Quantiva; Thermo Fisher) was used for the analysis in MRM mode. All the ion transitions and retention times were optimized using chemical standards. Tracefinder (Thermo Fisher, USA) was applied for metabolite identification and peak integration. The peaks were manually checked for the analysis. Pooled QC samples were inserted in the batch to ensure system stability.

For untargeted metabolomics, an Orbitrap mass spectrometer (QExactive; Thermo Fisher) was used for the analysis in DDA mode. An in-house database containing MS/MS spectra of over 1,500 metabolites was incorporated for metabolite identification. Tracefinder (Thermo Fisher, USA) was used for metabolite identification based on MS/MS fragment matching. Library score (LS) was applied to confirm the confidence of metabolite identification. Only metabolites with an LS of >30 were considered confirmed. Otherwise, they were assigned as a putative identification. The peaks were manually checked for the analysis. Pooled quality control (QC) samples were inserted in the batch to ensure system stability.

Normalization was performed before statistical analysis. The missing values were replaced with half of the minimum values in all the samples. Peak areas were normalized relative to the mean of the total area of a sample. Both targeted and untargeted metabolomics data were combined and imported into the R software (version 3.6.2) for multivariate analysis.

### Quantification of salivary cytokines using multiplexed bead immunoassay.

We collected 194 salivary samples at days −21, 0, 3, 7, and 28 from 40 subjects who were selected for quantification of inflammatory cytokines (Fig. 1a). All samples were subpacked (1.0 ml sample in a 1.5-ml Eppendorf tube) and stored at −80°C until measurements. Samples were thawed in an ice bath and vortexed, followed by centrifugation at 3,000 rpm for 5 min at 4°C. Supernatants were collected for further cytokine assays. Levels of the following 27 cytokines were analyzed using a BioPlex Pro human cytokine 27-plex assay kit (no. M500KCAF0Y; Bio-Rad, Hercules, CA, USA) in accordance with the manufacturer’s instructions: IL-1β, IL-1α, IL-2, IL-4, IL-5, IL-6, IL-7, IL-8, IL-9, IL-10, IL-12(p70), IL-13, IL-15, IL-17, eotaxin, basic FGF, granulocyte colony-stimulating factor (G-CSF), granulocyte-macrophage CSF (GM-CSF), IFN-γ, IP-10 (10-kDa IFN-γ-induced protein), monocyte chemoattractant protein 1 (MCP-1), MIP-1α, MIP-1β, platelet-derived growth factor BB (PDGF-BB), RANTES, tumor necrosis factor alpha (TNF-α), and vascular endothelial growth factor (VEGF). Mean fluorescence intensities of the 192 salivary samples and 8 standards were detected via a Luminex Flexmap 3D system (Luminex Corp., Austin, TX, USA). Cytokine concentrations were calculated by xPONENT build 4.2.1441.0 (Luminex Corp.) using a five-parameter fit algorithm. Values obtained from the reading of samples below the sensitivity limit of detection (LOD) or above the upper limit of the sensitivity method were interpolated using a cubic spline interpolation to calculate cytokine concentrations.

### Statistical analyses.

All statistical analyses were performed using R software (version 3.6.2). PCoA analysis on a range of distance metrics was performed in R using the vegan and ape packages. Quantifications of variance explained in plaque microbiome, metabolome, and salivary cytokine profiles were calculated using permutational multivariate analysis of variance (PERMANOVA) with the adonis function in the R package vegan (Fig. S1). The total variance explained by each variable was calculated independently of other variables and should thus be considered the total variance explainable by that variable. The differential abundance analyses of all measurement types were tested. First, an appropriate transformation/normalization method was applied: CLR transformation for microbial taxonomic profiles. The transformed abundances were then used to perform differential abundance analyses between time points or groups using custom R functions (available at https://github.com/shihuang047/crossRanger). To construct the co-occurrence network of molecular features from the multi-omics data sets, we identified significant associations between them using the Spearman correlation (|rho|>0.6; false discovery rate [FDR] *P < *0.05). The network was visualized in Cytoscape (version 3.7.1).

### Data availability.

The code and all the data sets used in this study are publicly available at http://mse.ac.cn/SoH.html.
