# Supplementary figures and images for: Longitudinal Multi-omics and Microbiome Meta-analysis Identify an Asymptomatic Gingival State That Links Gingivitis, Periodontitis, and Aging
Source: mBio. 2021 Mar 9;12(2):e03281-20. doi: 10.1128/mBio.03281-20 (PMC8092283; doi:10.1128/mBio.03281-20)

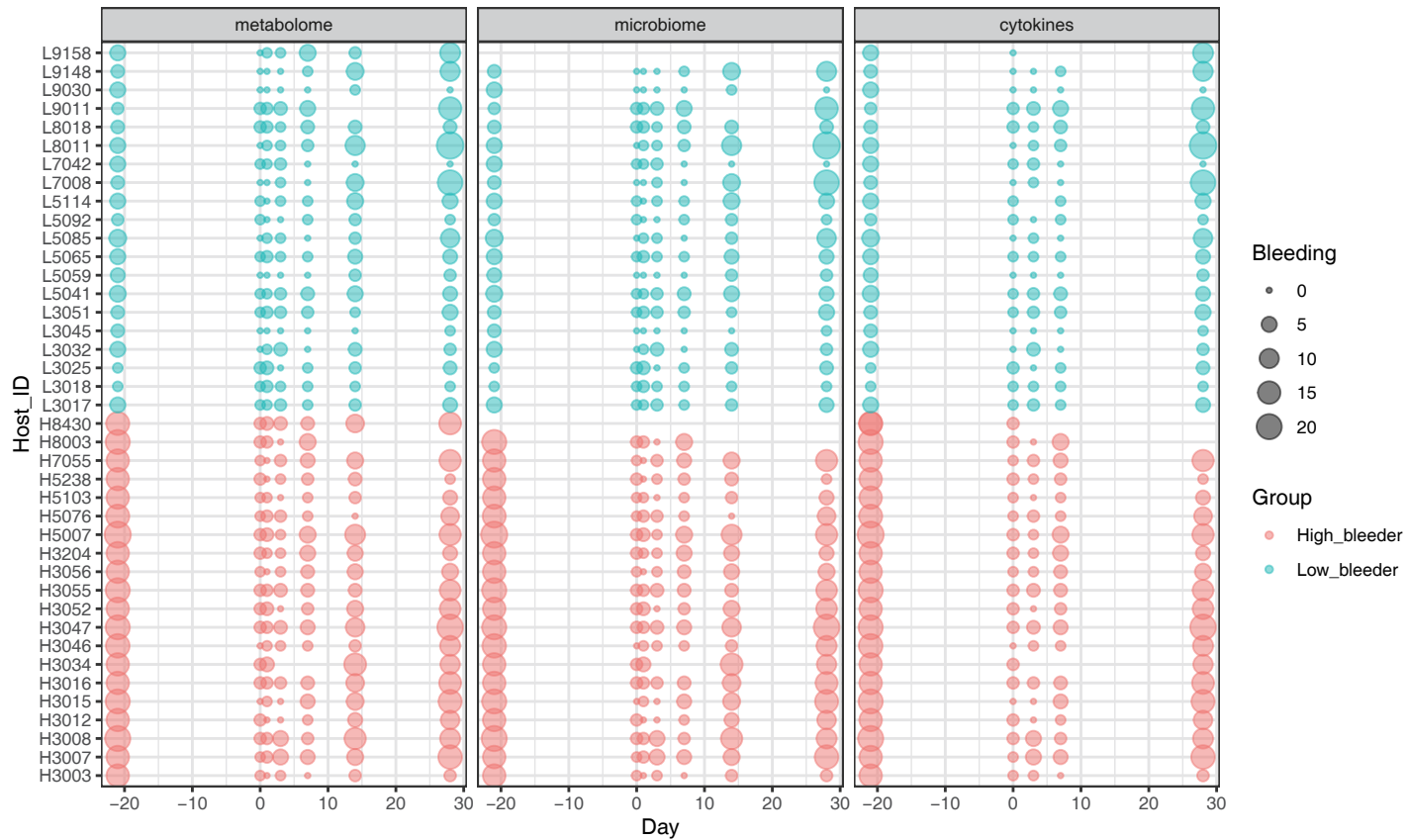

Supplement: FIG S1 [file mBio.03281-20-sf001.pdf]

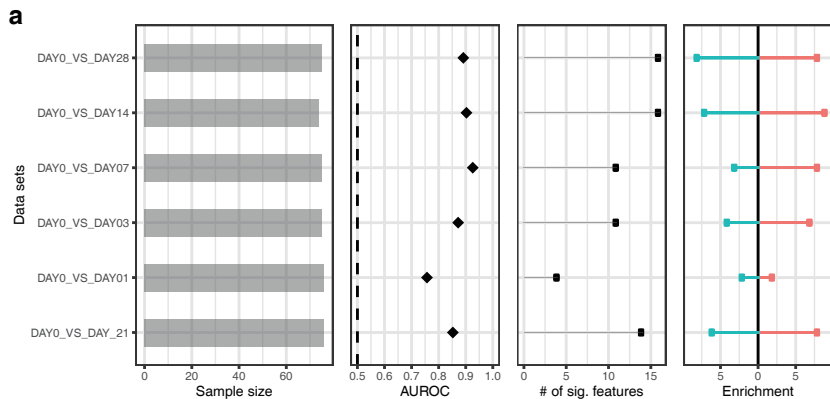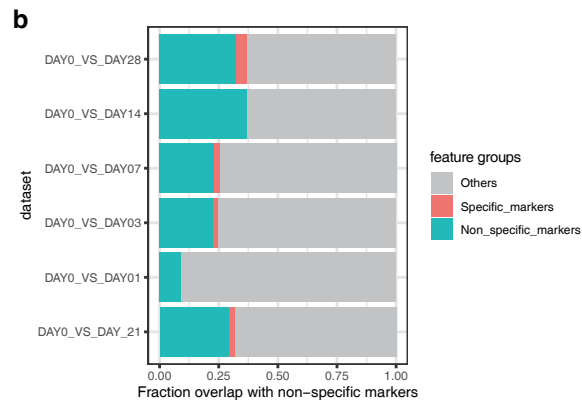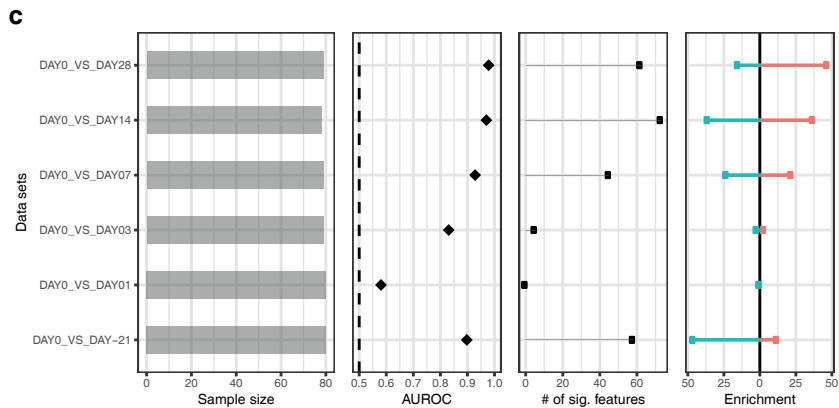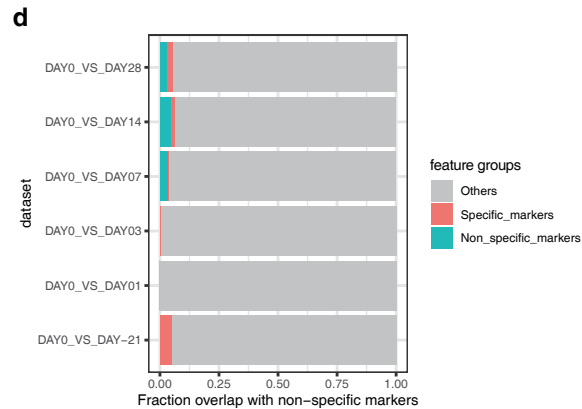

Supplement: FIG S2 [file mBio.03281-20-sf002.pdf]

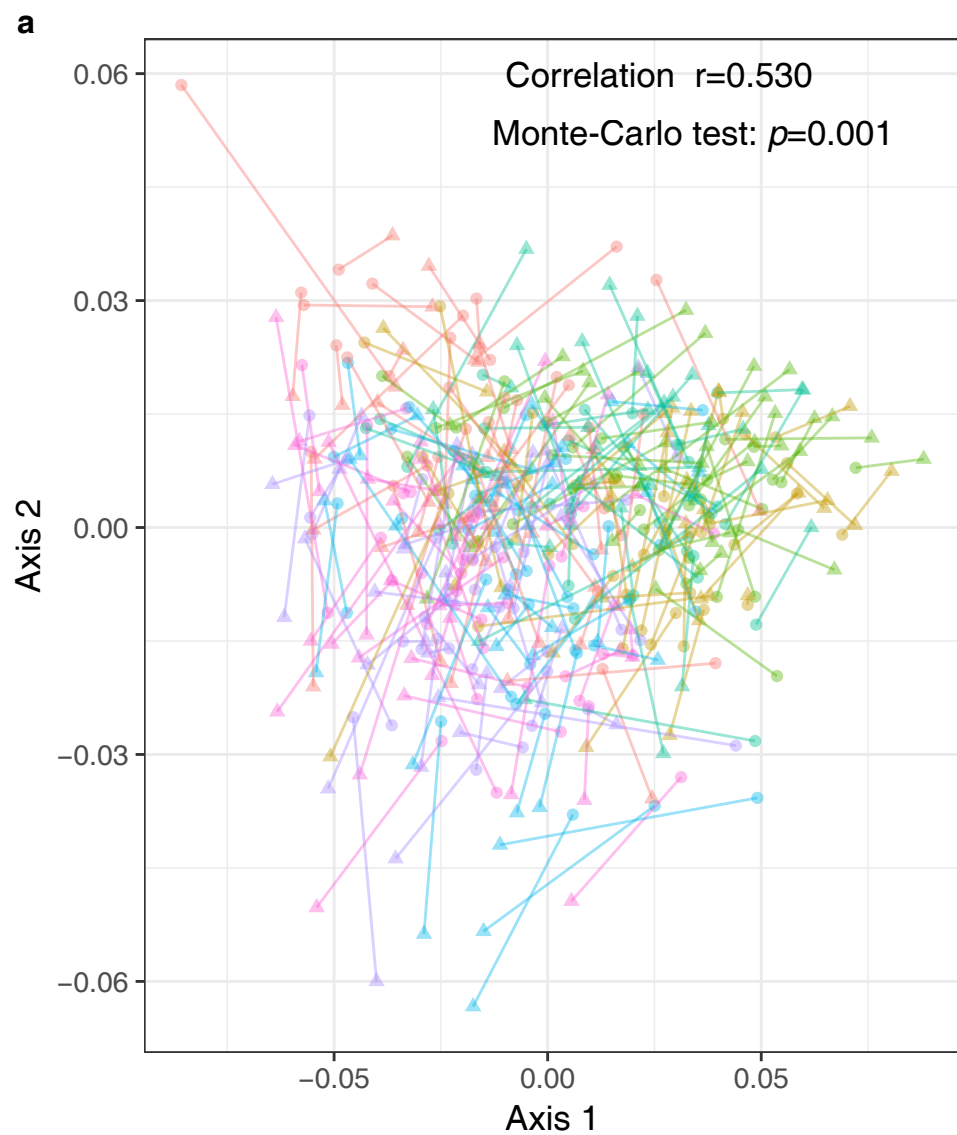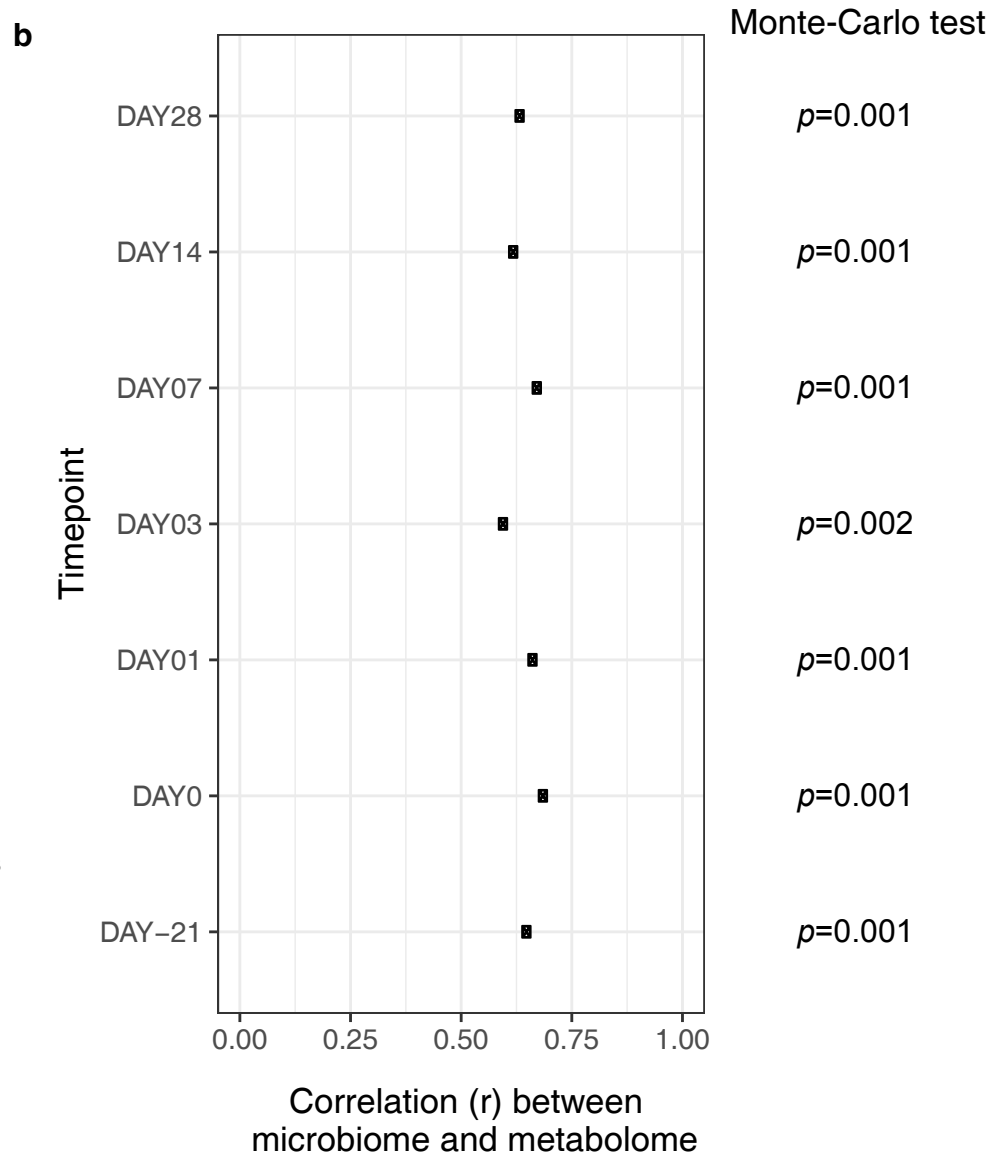

Supplement: FIG S3 [file mBio.03281-20-sf003.pdf]

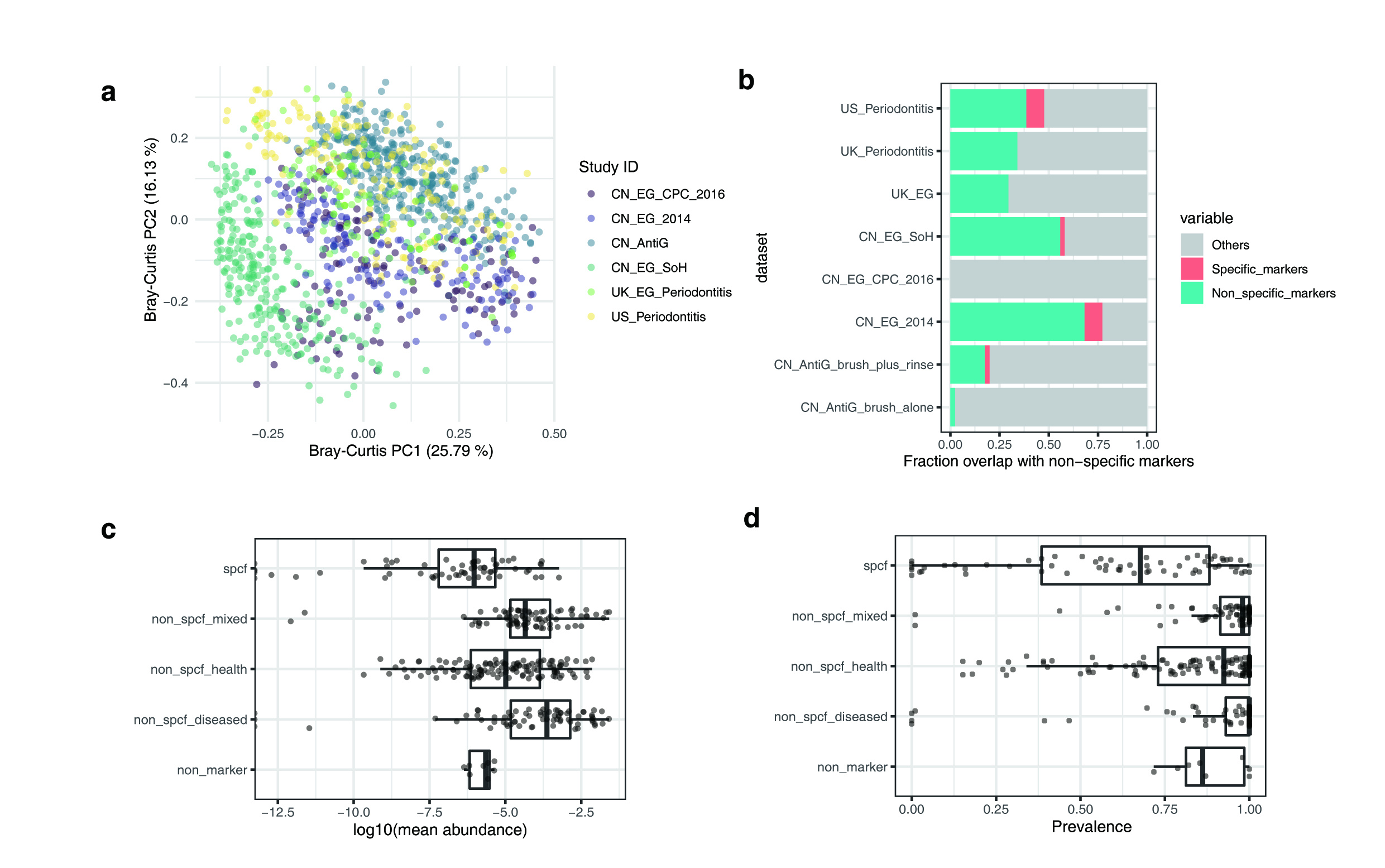

Supplement: FIG S4 [file mBio.03281-20-sf004.tif]
