# Supplementary material for: Longitudinal Multi-omics and Microbiome Meta-analysis Identify an Asymptomatic Gingival State That Links Gingivitis, Periodontitis, and Aging
Source: mBio. 2021 Mar 9;12(2):e03281-20. doi: 10.1128/mBio.03281-20 (PMC8092283; doi:10.1128/mBio.03281-20)
Supplement: FIG S5 [file mBio.03281-20-sf005.pdf]

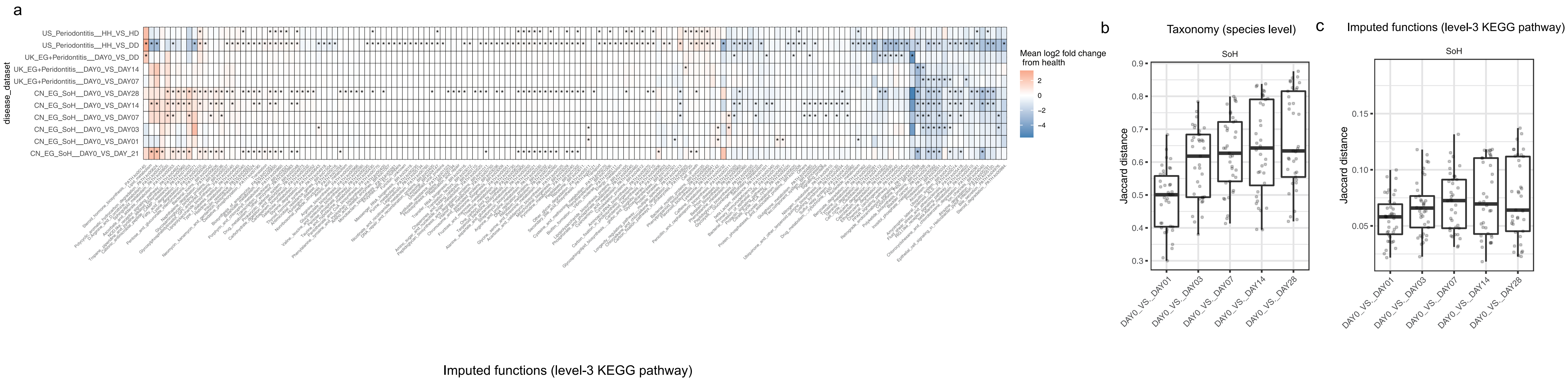
a
US_Periodontitis__HH_VS_HD
US_Periodontitis__HH_VS_DD
UK_EG+Peridontitis__DAY0_VS_DD
UK_EG+Peridontitis__DAY0_VS_DAY14
UK_EG+Peridontitis__DAY0_VS_DAY07
CN_EG_SoH__DAY0_VS_DAY28
CN_EG_SoH__DAY0_VS_DAY14
CN_EG_SoH__DAY0_VS_DAY07
CN_EG_SoH__DAY0_VS_DAY03
CN_EG_SoH__DAY0_VS_DAY01
CN_EG_SoH__DAY0_VS_DAY_21
disease_dataset
Mean log2 fold change from health
2
0
−2
−4
Imputed functions (level-3 KEGG pathway)
b
Taxonomy (species level)
SoH
Jaccard distance
0.9
0.8
0.7
0.6
0.5
0.4
0.3
DAY0_VS_DAY01
DAY0_VS_DAY03
DAY0_VS_DAY07
DAY0_VS_DAY14
DAY0_VS_DAY28
c
Imputed functions (level-3 KEGG pathway)
SoH
Jaccard distance
0.15
0.10
0.05
DAY0_VS_DAY01
DAY0_VS_DAY03
DAY0_VS_DAY07
DAY0_VS_DAY14
DAY0_VS_DAY28
