# Supplementary material for: Longitudinal Multi-omics and Microbiome Meta-analysis Identify an Asymptomatic Gingival State That Links Gingivitis, Periodontitis, and Aging
Source: mBio. 2021 Mar 9;12(2):e03281-20. doi: 10.1128/mBio.03281-20 (PMC8092283; doi:10.1128/mBio.03281-20)
Supplement: TABLE S2 [file mBio.03281-20-st002.pdf]

Table S2. Overview of the 40-individual cohort that underwent the controlled oral hygiene regimen. (**a**) Overview of the cohort. (**b**) Background information on each subject that participated in the cohort.

**Demographic/Statistic Category**	**High_bleeder (n=20)**	**Low bleeder (n=20)**	**Overall (n=40)**	***p*-value**
**Age (Years)**				
Mean (SD)	33.60 (6.52)	37.65 (9.44)	35.63 (8.27)	0.123[a]
Min.-Max.	23 - 52	22 - 53	22 - 53	
**Gender**				
Female[b]	16 (80%)	16 (80%)	32 (80%)	1.000[c]
Male[b]	4 (20%)	4 (20%)	8 (20%)	
**Smoking**				
Yes[b]	2 (10%)	2 (10%)	4 (10%)	1.000[c]
No[b]	18 (90%)	18 (90%)	36 (90%)	

[a] Two-sided ANOVA *p*-value for the treatment comparison.

[b] The number (percent) of subjects in each category.

[c] Two-sided chi-square *p*-value for the treatment comparison.

[d] Two-sided Fisher's exact test *p*-value for the treatment comparison.

**Host_ID**	**Group**	**Age**	**Gender**	**Smoking**
3003	High_bleeder	38	F	N
3007	High_bleeder	37	F	N
3008	High_bleeder	29	F	N
3012	High_bleeder	30	F	N
3015	High_bleeder	33	F	N
3016	High_bleeder	30	F	N
3017	Low_bleeder	28	F	N
3018	Low_bleeder	22	F	N
3025	Low_bleeder	41	F	N
3032	Low_bleeder	53	F	N
3034	High_bleeder	52	F	N
3045	Low_bleeder	46	M	N
3046	High_bleeder	31	M	Y
3047	High_bleeder	31	F	N
3051	Low_bleeder	36	F	N
3052	High_bleeder	37	M	N
3055	High_bleeder	34	M	N

3056	High_bleeder	28	F	N
3204	High_bleeder	23	F	N
5007	High_bleeder	36	F	N
5041	Low_bleeder	41	F	N
5059	Low_bleeder	47	F	N
5065	Low_bleeder	31	F	N
5076	High_bleeder	33	F	N
5085	Low_bleeder	49	F	N
5092	Low_bleeder	31	M	Y
5103	High_bleeder	31	F	N
5114	Low_bleeder	31	F	N
5238	High_bleeder	47	F	N
7008	Low_bleeder	46	F	N
7042	Low_bleeder	47	F	N
7055	High_bleeder	30	F	N
8003	High_bleeder	30	M	Y
8011	Low_bleeder	30	F	N
8018	Low_bleeder	23	M	N
8430	High_bleeder	32	F	N
9011	Low_bleeder	29	M	Y
9030	Low_bleeder	31	F	N
9148	Low_bleeder	47	F	N
9158	Low_bleeder	44	F	N

6

7
