# Supplementary material for: Longitudinal Multi-omics and Microbiome Meta-analysis Identify an Asymptomatic Gingival State That Links Gingivitis, Periodontitis, and Aging
Source: mBio. 2021 Mar 9;12(2):e03281-20. doi: 10.1128/mBio.03281-20 (PMC8092283; doi:10.1128/mBio.03281-20)
Supplement: TABLE S1 [file mBio.03281-20-st001.pdf]

Table. S1. Clinical study design. This is a single-leg, examiner-blind and randomized clinical study. Forty healthy adult volunteers who meet the inclusion criteria were recruited for this study. The procedures taken at each time points along 48-day NG-Baseline-EG process were highlighted via ‘X’ below. See **Methods** and **Fig. 1a** for additional details.

Phase	Oral Hygiene			Induced Gingivitis						Recovery
Procedure	Day -21	Day -14	Day -7	Day 0	Day 1	Day 3	Day 7	Day 14	Day 28	Follow-up
Informed Consent	X									
Medical History	X									
Demographics	X									
Inclusion/Exclusion	X									
Continuance Criteria		X	X	X	X	X	X	X	X	
saliva	X			X	X	X	X	X	X	X
Periodontal Examination	X									
Mazza Gingival index	X	X	X	X	x	x	X	X	X	X
Gingival plaque collection	X			X	X	X	X	X	X	
Dental Prophylaxis	X	X	X							
Tooth polishing	X		X							X
Daily Supervised Oral Hygiene	X	X	X	X	X	X	X	X		X
Adverse Event Recording	X	X	X	X	X	X	X	X	X	
Subject Accountability									X	
